# Novel Nanosized Chitosan-Betulinic Acid Against Resistant Leishmania Major and First Clinical Observation of such parasite in Kidney

**DOI:** 10.1038/s41598-018-30103-7

**Published:** 2018-08-06

**Authors:** Tahereh Zadeh Mehrizi, Mehdi Shafiee Ardestani, Mostafa Haji Molla Hoseini, Ali Khamesipour, Nariman Mosaffa, Amitis Ramezani

**Affiliations:** 1 Department of Clinical Research, Pasteur Institute of Iran, Tehran, Iran; 2 Department of Radiopharmacy, Faculty of Pharmacy, Tehran University of Medical Sciences, Tehran, Iran; 3 Department of Medical Immunology, School of Medicine, Shahid Beheshti University of Medical Sciences, Tehran, Iran; 4 Center for Research and Training in Skin Diseases and Leprosy, Tehran University of Medical Sciences, Tehran, Iran

## Abstract

Regarding the antiparasitic effects of Betulinic acid (B) against Leishmaniasis, it was loaded into nanochitosan (K) for the first time in order to improve its therapeutic effects and decrease its side effects for the treatment of Leishmania major-infected Balb/c mice. Improvement the therapeutic efficacy of Bas an anti-leishmania agent through increasing the effective dose was achieved by using a novel solvent and phase separation method for K synthesis. The synthesized K with the size of 102 nm and Betulinic acid-nanochitosan (BK) with the size of 124 nm and drug loading efficiency of 93%, cellular uptake of 97.5% with the slow drug release pattern was prepared. To increase the therapeutic dose, a modified 10% acetic acid solvent was used. The *in vitro* and *in vivo* results showed that the nanodrug of BK was non toxic by 100% and BK20 mg/kg could completely performed the wound healing and inhibit the parasite in a large extent (P ˂ 0.001) compared to other groups. Therefore, BK could be considered as an alternative regimen for treatment of L. major.

## Introduction

Cutaneous leishmaniasis is caused by a number of types of protozoan parasites of the genus leishmania^1^. The disease is the most common form of leishmaniasis and causes scars on the body for lifetime and is the most common infectious disease worldwide^2^.

Chemotherapeutic agenst including glucantime (as the choise treatment regimen), Miltefosine, Amphotericin B and paromomycin are used for treatment of cutaneous leishmaniasis and their clinical use is restricted due to their side effects, toxicity and development of drug resistance^3^. Therefore, the global approach is toward to introduce the new treatment regimens for this disease^4^. In this regard, Betulinic acid (B) is recently introduced as an antileishmanial compound^5,6^. B is a triterpenoid pentacyclic compound with several biological properties including antiparasitic activity. B is a natural material which can be obtained from several different types of plants such as *P.andrieeuxii* and betulin as a metabolic precursor^7^. Triterpenoids induce apoptosis through several different mechanisms. B induces apoptosis through direct disruption in mitochondrial function, changes in the expression levels of Bcl-2 protein family and by activating the NF-κB^8^.

Recently, some studies showed that betulin heterocyclic derivatives including B have antiparasitic activity against Leishmania donovani^9–11^. It has been demonstrated that B is able to induce apoptosis through inhibition of DNA topoisomerase I and II activity in L. donovani^11,12^. However, clinical use of B is limited due to its poor solubility and relatively short plasma half-life^13^.

In this regard, to dissolve these problems, researchers have used of nanocarriers. One of the most proved functions of nanocarriers is increasing the drug solubility, decreasing the drug toxicity andtargeted drug delivery and also nanocarriers are biodegradable and biocompatible^14,15^. In this way, one of the nanocarrier is nanochitosan (K).

K is introduced as one the nanocarriers and such as chitin has antileishmanial effects^16^, increase the drug solubility at proper pH and is effective in wound healing by its own^17^.

K properties are included targeted drug delivery^16,18^, activates macrophage and induces cell mediated immunity, increasing the cellular uptake and slowing the drug release due to the positive surface charge resulting in prolongs the retention time of drugs and continuous drug release *in vivo* as well as improve drug bioavailability^19^. One of the other reason that makes K as a slow drug release carrier is its acid-resistive characteristic^17^.

K synthesis methods are included solvent evaporation, ionic cross-linking, spray-drying ionic gelation, covalent cross-linking, emulsion cross-linking, polymerization, self-assembly, coacervation/precipitation, emulsion-droplet coalescence method, spray-drying, reverse micellar method, precipitation and sieving methods^19^. Phase separation technique used in the present study is a subclass of precipitation method. It is the first study reported using novel safe solvent with enhanced solubility for B, using phase separation method for synthesizing of Betulinic acid-nanochitosan (BK) and using high dose of B for cure of cutaneous leishmaniasis in Balb/c mice without any side effect and toxicity.

Moreover, the penetration depth of the parasite was detected in the kidney of mice in control group which is reported for the first time. B was loaded into K and afterthat the toxicity and efficacy of the formulation were evaluated against L. major *in vitro* and *in vivo* environment and the *in vivo* results were confirmed by histopathological studies including parasite number and pathological effects.

## Results

### Size, size distribution and zeta potential of nanoparticles

K was synthesized with the size of 102 nm, Polydispersity Index (PDI) of 0.2 and zeta potential of 14 mV. Also, nanodrug BK with the size of 112 nm, PDI of 0.3 and zeta potential of 8 mV was synthesized. The observed difference in PDI from 0.2 in K to 0.3 in nanodrug indicated occurrence of a novel aggregate in nanodrug which in turn indicated the drug loading into K^20^.

### Drug loading efficiency

The drug loading efficiency was calculated by using the standard curve which was equal to 93%. In other words, 93% of primary drug used were loaded into K.

### Thin layer chromatography (TLC)

The Retardation Factor (R_f_) results of TLC for B was 0.84, K = 0.36 and BK was equal to 0.26. Therefore, based on these results, it was found that the three compounds were pure.

### X-ray photoelectron spectroscopy (XPS) analysis

**Figure 1:**
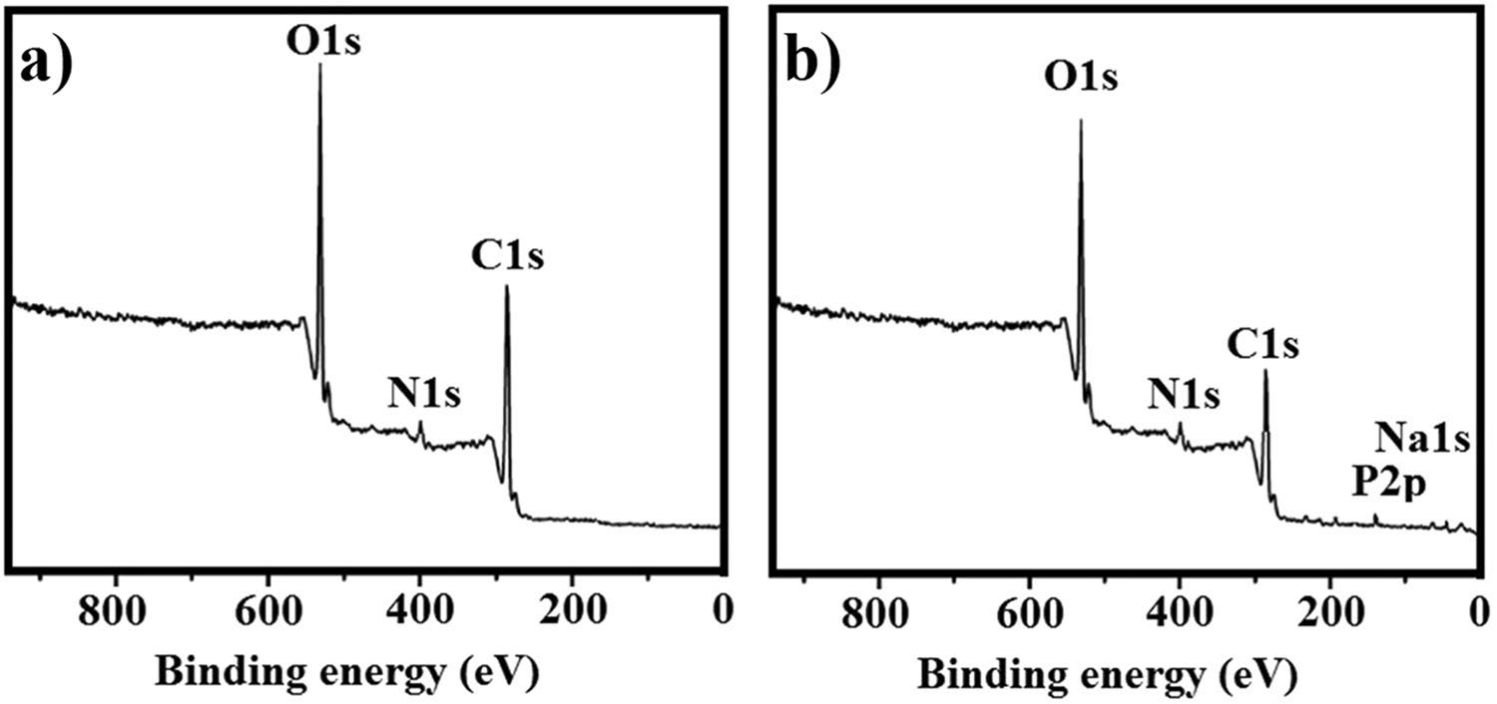
XPS spectra of (**a**): K before drug loading and (**b**): K after B loading. The related peaks of K and BK indicate loading of B into K.

XPS analysis is a quantitative spectroscopic technique for measure the elemental composition of materials. There are three main peaks in XPS analysis related to C, O and N elements: 284–289 for C, 400 for N and 531 eV for O^21,22^. The results of XPS analysis showed loading of B into K (Fig. 1).

### Morphology evaluation by using Scanning Electron Microscopy (SEM)

**Figure 2:**
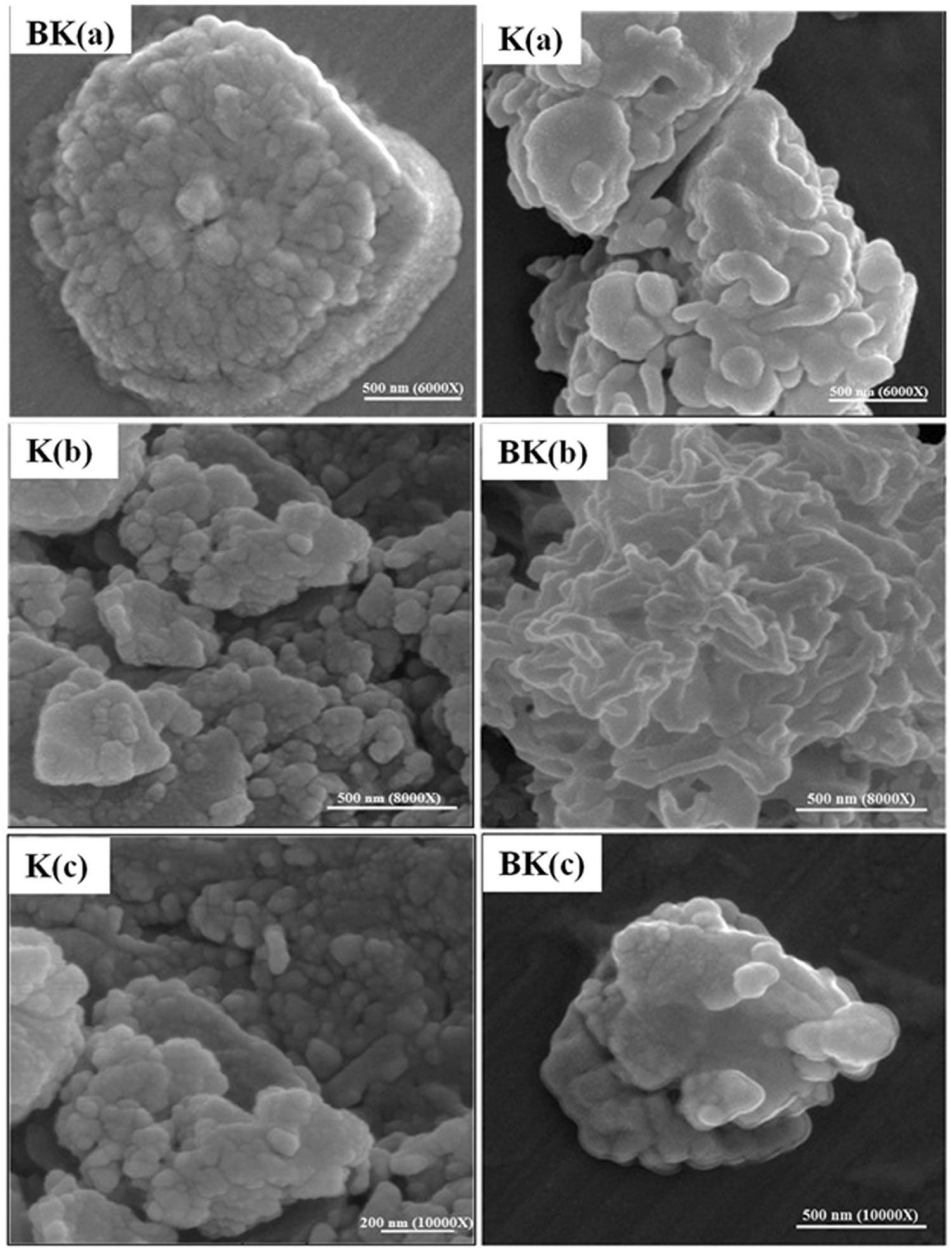
SEM micrographs of K and nanodrug BK with magnification of (**a**): 6000X; (**b**) 8000X and (**c**): 10000X.

The results of SEM indicated synthesis of K and BK. K was formed spherical, while nanodrug BK was more intended to be elliptical and swollen compared to the K. This change formation results from drug loading into nanoparticles (Fig. 2).

### Morphology evaluation by Atomic Force Microscopy (AFM)

**Figure 3:**
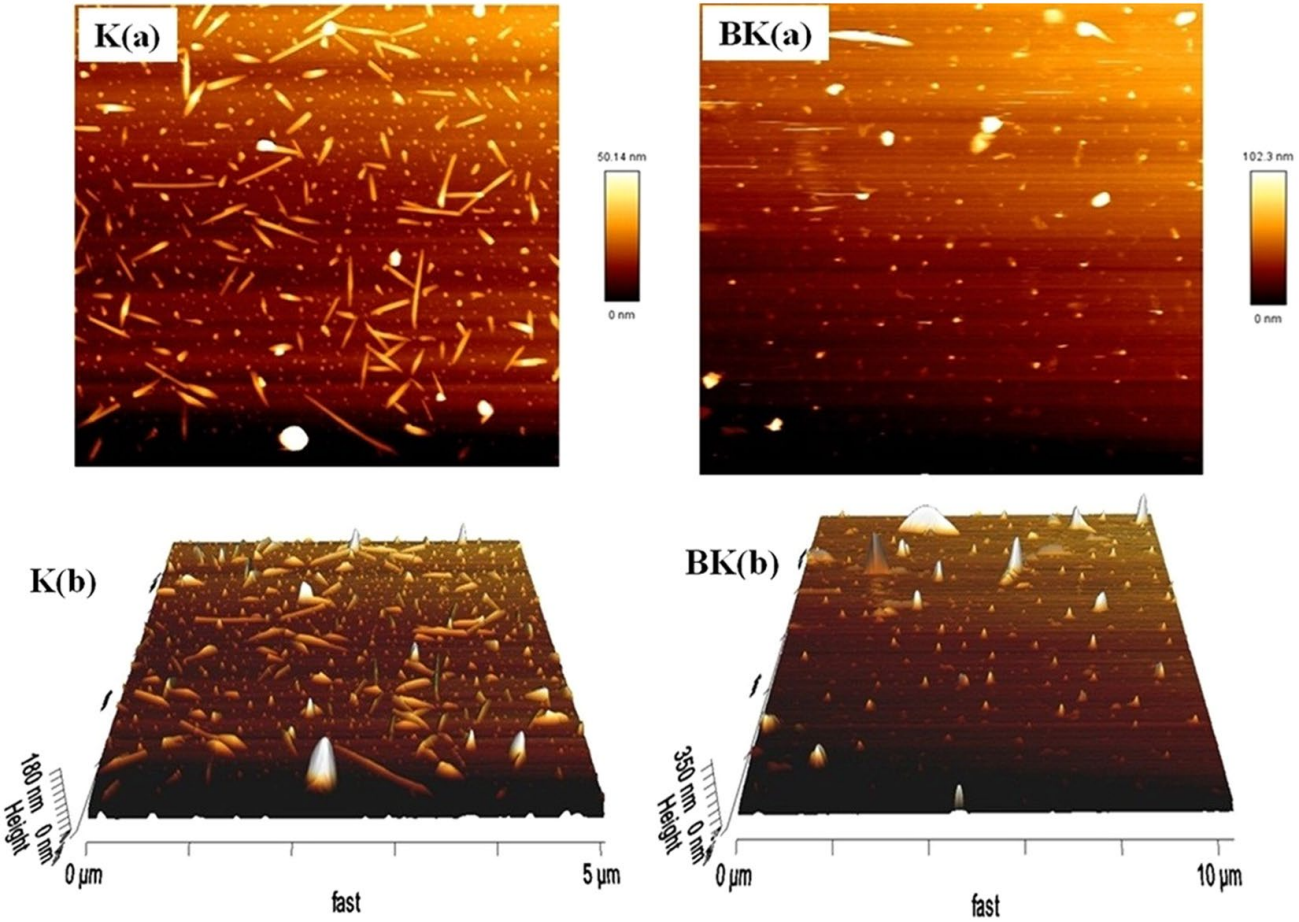
The (**a**): two and (**b**): three dimensional of AFM micrographs for K and nanodrug BK.

Due to the special process used for sample preparation in AFM microscopy (the lyophilized sample was directly placed on a grid and lack of sample dispersion and sonication), the K was formed as needle-shaped and BK as more swollen compared to K, indicating the drug loading into K (Fig. 3).

### Morphology evaluation by Transmission Electron Microscopy (TEM)

**Figure 4:**
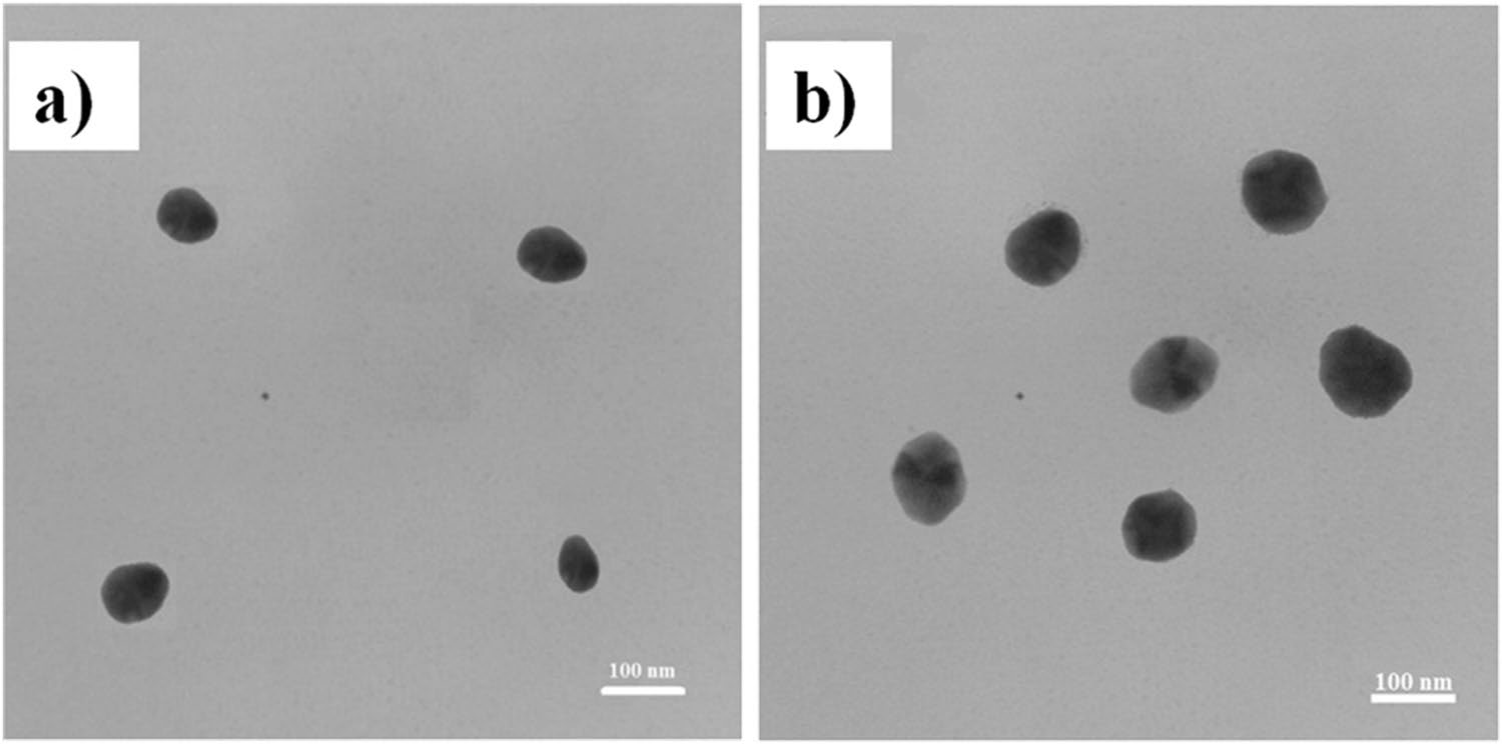
TEM micrographs of (**a**): K and (**b**): nanodrug BK. The scale bar is equal to 100 nm.

The observed spherical forms of nanoparticles in TEM is resulted from sample dispersion and sonication processes. The results showed that K was formed as small circle and nanodrug was found to be as bigger circle indicating the drug loading into nanoparticles (Fig. 4).

### Fourier-Transform Infrared Spectroscopy (FTIR)

**Figure 5:**
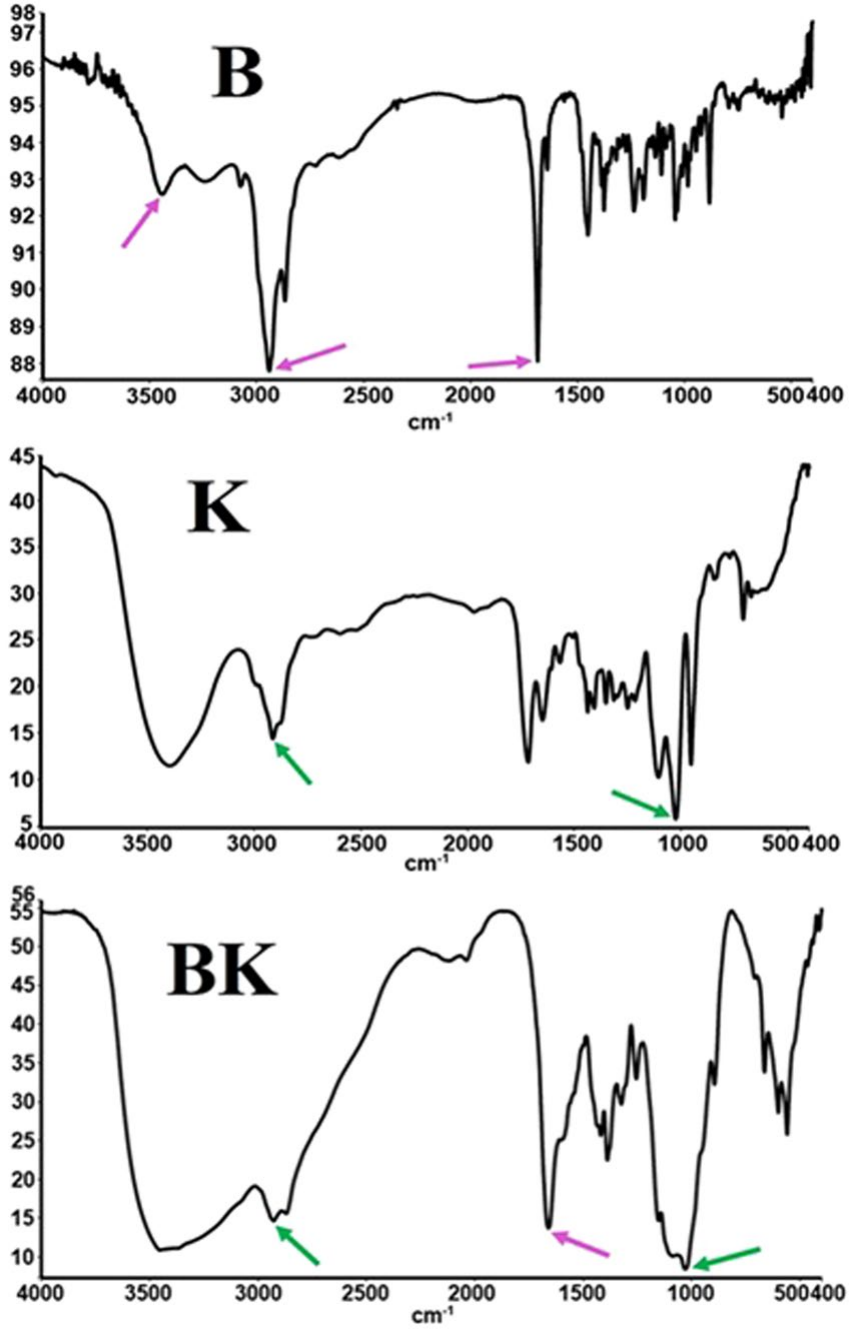
FTIR spectrum of B (red arrows), K (green arrows) and nanodrug BK. As the picture shows, B (shows by arrows) preserves its related FTIR spectrum in nanodrug BK structure. Therefore, the results showed that B was loaded into K physically.

The FTIR spectrum from drug indicated the presence of B (regions of 2900–3500 cm^-1^ for OH and CH groups and 1700 cm^-1^ for C=O group) into K nanoparticles (regions of 2900–3500 cm^-1^ for NH2 and OH groups and 1000–1200 cm^-1^ for C=O group). The FTIR spectrum for BK was as 2800–3500 cm^-1^ for OH and NH2 of B and K, 2900–3000 for NH2 of K and 1000–1200 for C=O of K, 1700 for carbonic group of B. In other words, the drug was physically loaded onto particles (Fig. 5).

### Proton Nuclear Magnetic Resonance (H-NMR)

**Figure 6:**
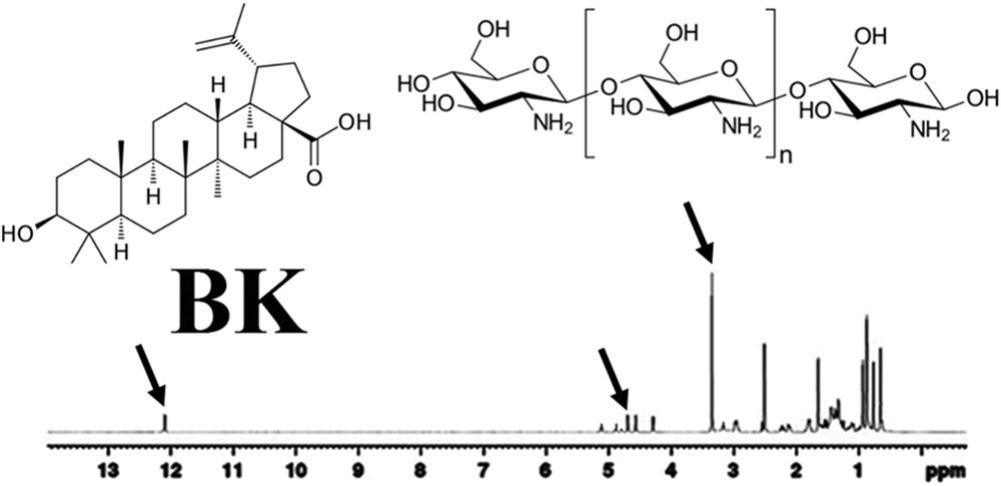
H-NMR spectrum of BK. The related arrows showsthe CH-O group of K, C = CH and carbonic acid groups of B, indicating that B was loaded into K.

H-NMR study was performed for Nanodrug BK. The observed spectrums and related groups were found as follow: The spectrum of 1–2 was related to CH of K and B, 2–3 for NH2 and OH groups of K and B. Also, the spectrum of 3–4 was related to CH-O of K, 4–5 to C=CH of B and 12 was related to the carbonic acid of B. These findings assured us that B was loaded into K physically (Fig. 6).

### Drug release study

**Figure 7:**
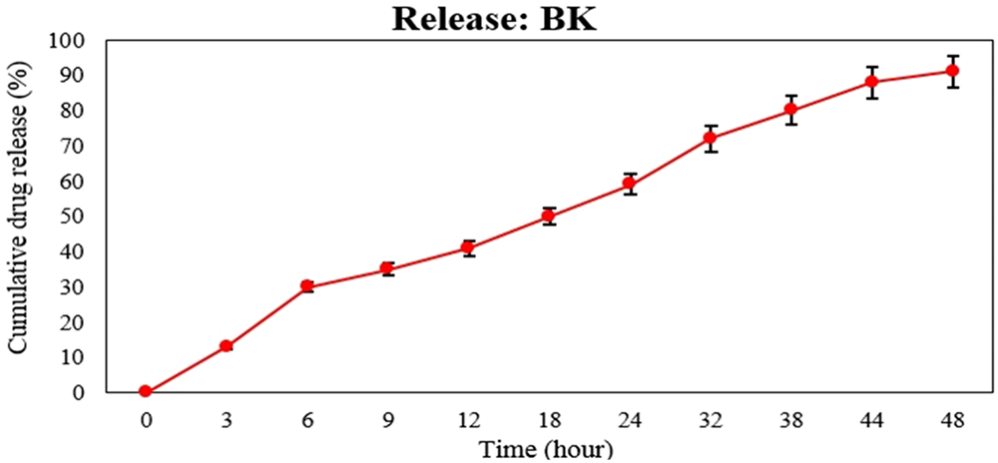
The cumulative release curve of B from K. As the graph shows, the pattern of drug release from nanoparticles follows the slow release pattern.

The results of drug release indicated that K had a good ability for drug retention and the drug was released from this nanostructure in a sustained and slow manner. In the first six hours of the study, 30% of the loaded drug was released and 90% of loaded drug was released in the time of 48 h. The drug release study was optimized at 37 °C. Therefore, BK in drug delivery systems is a controlled drug release device (Fig. 7).

### Cellular uptake of nanodrug BK20 µg/ml by using flowcytometry

**Figure 8:**
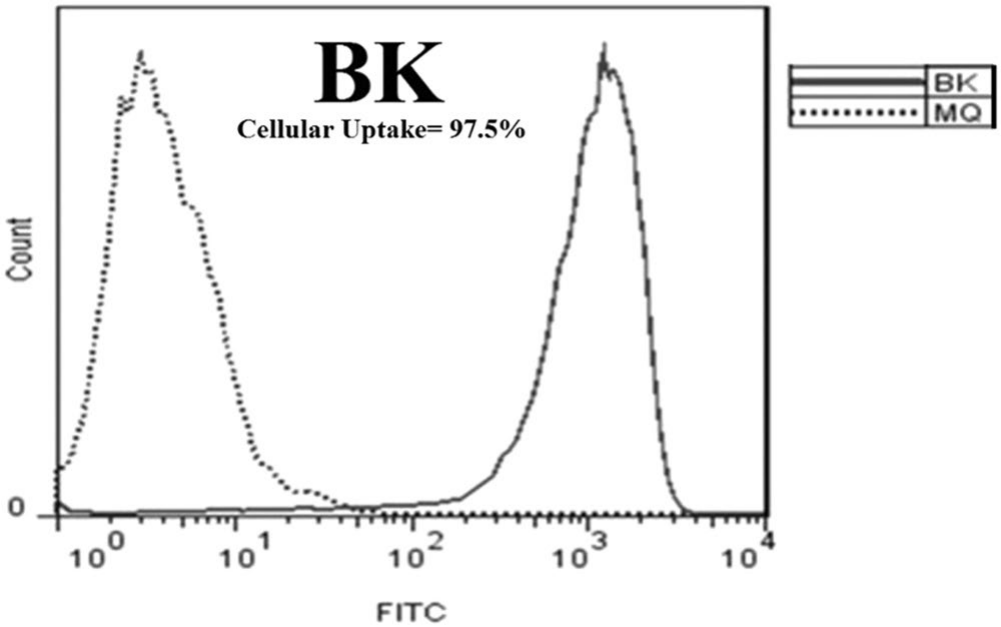
The flow cytometry results related to cellular uptake of nanodrug BK into macrophages with the cellualr uptake of 96.8%.

Cellular uptake of B-loaded chitosan nanoparticles at the drug concentration of 20 µg/ml (nanodrug BK20) (µg/ml and mg/kg were used for *in vitro* and *in vivo* environments, respectively) was evaluated by flow cytometry method. Regarding the (Fig. 8), the cellular uptake of nanodrug BK20 was found to be 97.5%. Therefore, loading of B onto K considerably increased its cellular uptake.

### Cellular uptake of formulations by using fluorescent microscopy

**Figure 9:**
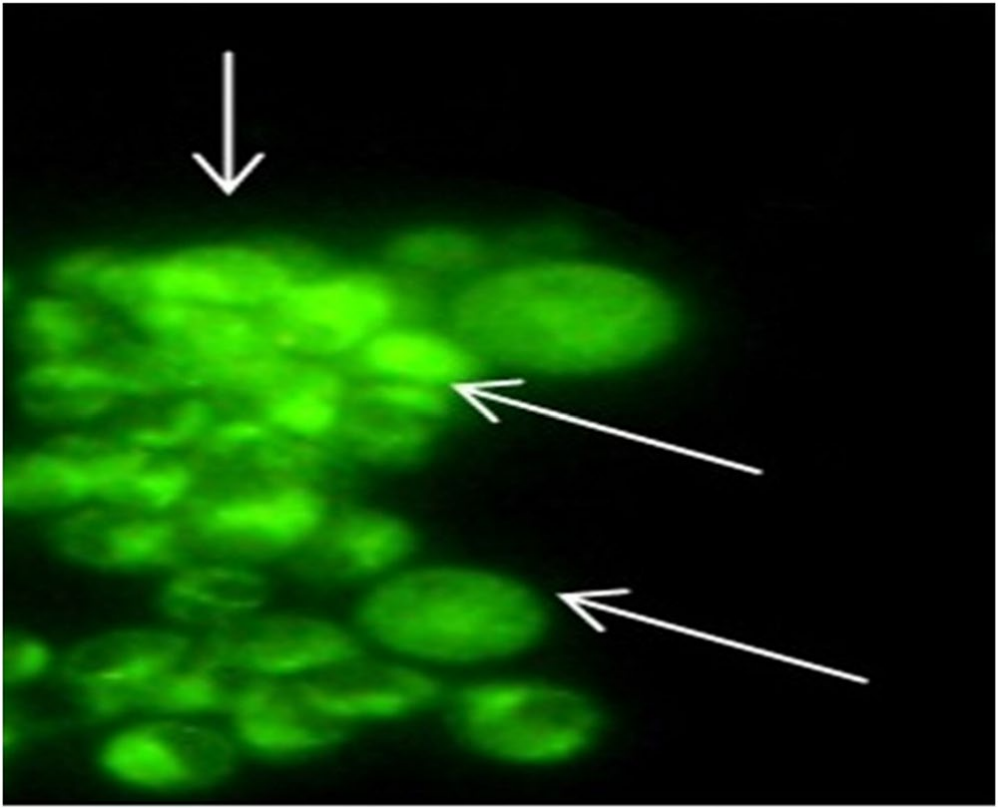
Cellular uptake of nanodrug BK into macrophages evaluated by Fluorescent microscopy.

Due to the low water solubility of B and its low cellular uptake, it is possible to directly trace its cellular uptake when loaded into K by using fluorescent microscopy. Figure 9 shows the peritoneal macrophages after 4 h incubation with BK20. As the fluorescent image shows, BK was uptaken by macrophage cells very well.

### *In vitro* evaluation the viability of nanodrug BK

**Figure 10:**
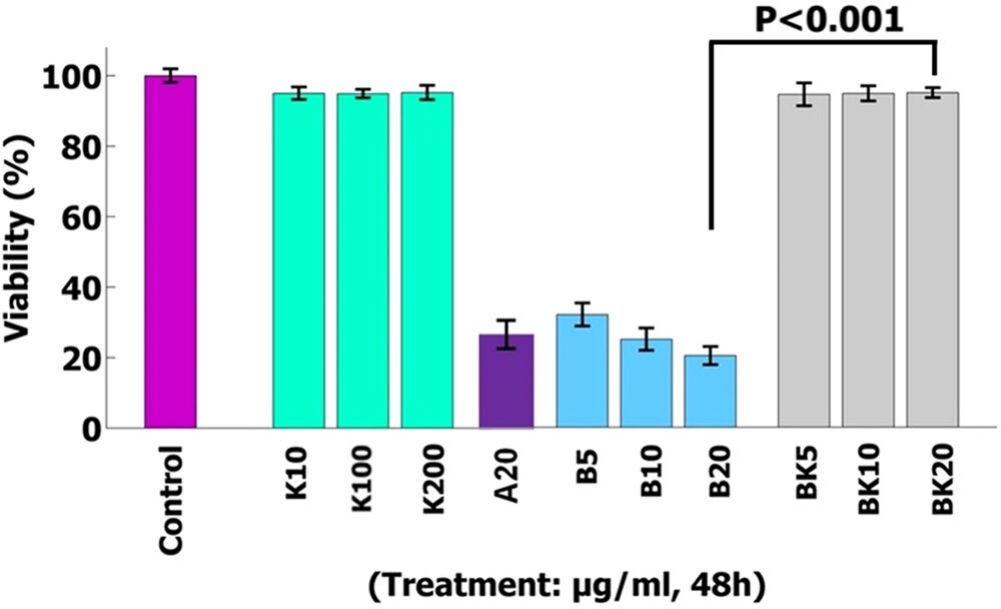
The viability effects showed that BK20 µg/ml and K10 µg/ml were perfectly non-toxic on the peritoneal macrophage compared to control group (P < 0.001). The values are expressed as Mean ± SD from three independent experiments.

At first, the macrophage viability was found to be 97% by using trypan blue exclusion test. The viability results showed that nanodrug BK20 (20 µg/ml) and K10 µg/ml were perfectly non-cytotoxic (by 100%) compared to the negative control group (peritoneal macrophages healthy) and positive control group (A20 µg/ml group) (P < 0.001) (Fig. 10). The viability was evaluated in the three incubation times of 12, 24 and 48 h and due to similarity of the results, the viability of 48 h was reported here. Since K had no toxicity by its own and is effective to reduce the drug toxicity, therefore when B was loaded into K, the nanodrug toxicity was decreased by 100%. The solvent used for viability evaluation was Phosphate Buffered Saline (PBS).

### Killing effects of the nanoformulations on the promastigote

**Figure 11:**
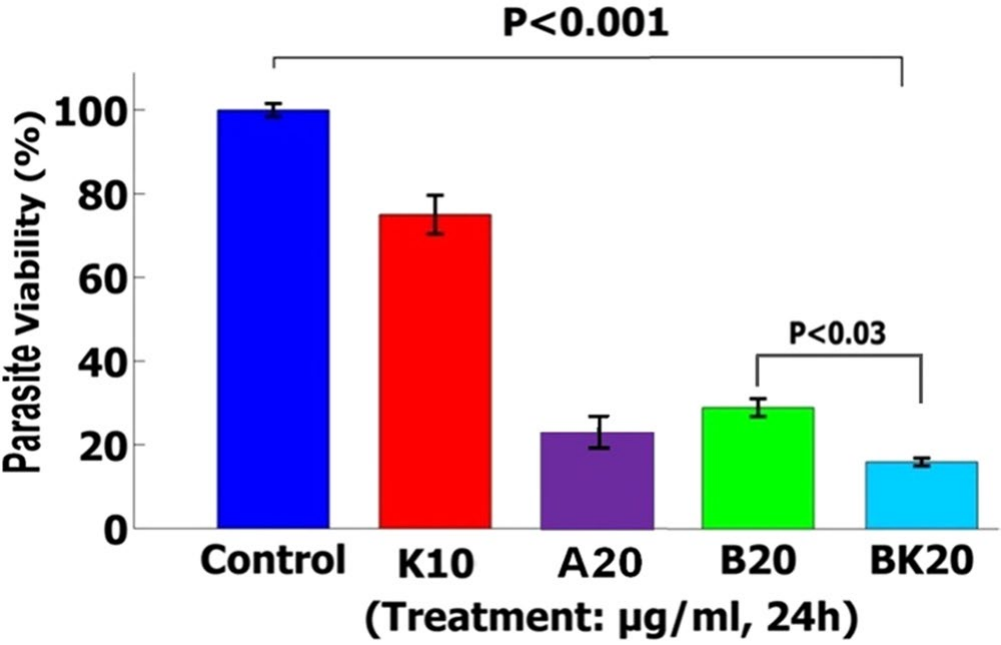
The killing effects showed that nanodrug BK20 µg/ml significantly killed 86% of L. major promastigotes compared to the control group (non-treated promastigotes) after 48 h incubation (P < 0.001). The values are expressed as Mean ± SD from three independent experiments.

The results showed that BK20 (20 µg/ml) was effective to killing the parasite by 86% compared to negative control group (L. major promastigotes) (P < 0.001) and positive control group (A20 µg/ml) (Fig. 11). In this test, the killing effects of the nanodrug were evaluated against the parasite, therefore reducing the viability indicated the enhancement of the parasite killing effects which in turn indicated the effective killing effects of nanodrug on promastigote of parasite. The solvent used for evaluation the killing effects of formulations was PBS.

### Inhibition effects of the nanoformulations on the amastigotes

**Figure 12:**
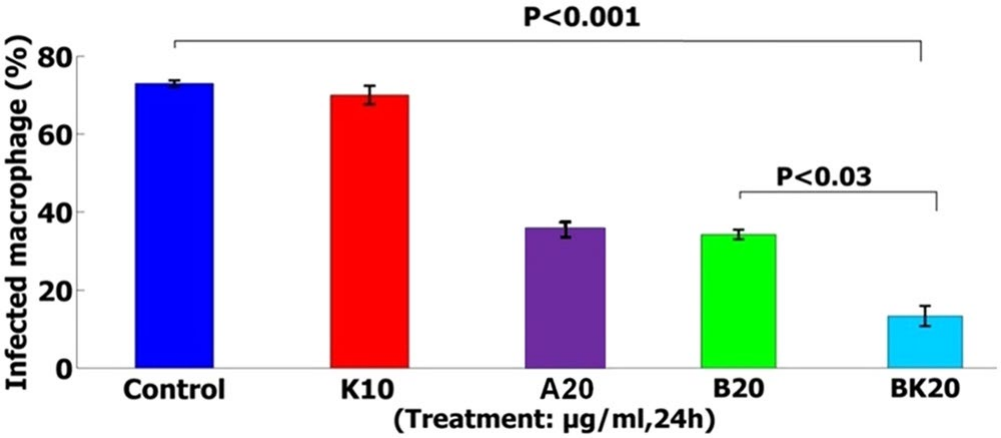
The inhibition effects showed that nanodrug BK20 µg/ml significantly inhibits the intracellular parasite by 81% compared to the control group (peritoneal macrophage) after 48 h incubation (P < 0.001). The values are expressed as Mean ± SD from three independent experiments.

Initially, the infection rate and mean number of amastigotes per a macrophage were found to be 73 ± 1% and 7 ± 1, respectively. The evaluation results of inhibition effects showed that nanodrug BK20 µg/ml was success to inhibit the amastigotes (L. major infected macrophages) by 81% compared to the negative control group (infected macrophages) (P < 0.001) and positive control group (A20 µg/ml group) (Fig. 12). Therefore, the more potent nanodrug, the more parasite inhibition effects were achieved and as a result the viability was decreased. The solvent used for evaluation the inhibition effects of formulations was PBS.

### Nitric oxide (NO) generation assay

**Figure 13:**
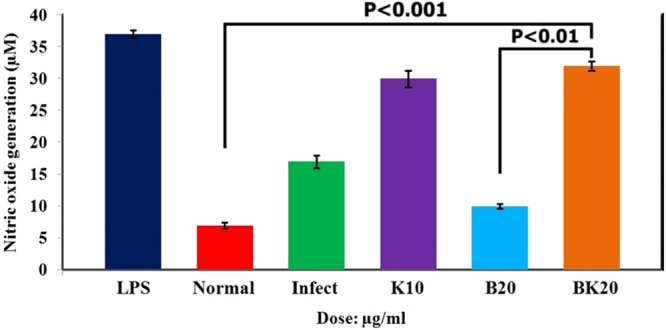
NO production by pertoneal macrophages (normal and L.major infected, both as negative control) against LPS (positive control), K10, B20 and BK20 µg/ml stimulation. Therefore, BK and K significantly increased the NO production compared to B (P < 0.01) and negastvie control groups (P < 0.001). Data arepresented as the mean ± SD in triplicate. (LPS: Lipopolysaccaride).

NO generation by infected macrophages is a symptom of macrophage activation to kill the intracellular parasites. The results of NO showed the potency of BK20 µg/ml and K10 µg/ml to produce NO compared to the free drug. In other words, infected macrophages incubated with the BK and K produced higher NO concentration compared to infected macrophages incubated with B (P < 0.001) (Fig. 13). In this experiment, LPS was regarded as positive control group, and healthy macrophages (normal negative control group) as well as L. major infected macrophages were regarded as negative control group.

### The solvent design

To prepare a desire solvent, a panel of different solvents including dimethyl sulfoxide (DMSO) (10% v/v), methanol (7% v/v), ethanol (9% v/v), bile salt (2% w/v), NaOH (1% w/v), tween 80 (1% v/v), acetic acid (0–100% v/v) was provided. The solvents were evaluated under different concentrations, pH, temperature and agitation time. Finally, the best solvent was 10% acetic acid at pH 6.5, temperature 56 °C after 48 h agitation. The pH of solvent was modified by using 10 N NaOH. The solvent was suitable to increase the solubility of B by 300 folds and prepare the drug dose of 20 mg/kg.

### Drug solubility *in vivo* environment

In the novel solvent, the following finding was observed: amount of dissolved drug in delivery system was 6 mg/ml with the drug solubility rate of 0.02 µg/ml, and as a result BK’s enhanced solubility by 300 folds was achieved.

### *In vivo* results of the nanoformulations toxicity

**Figure 14:**
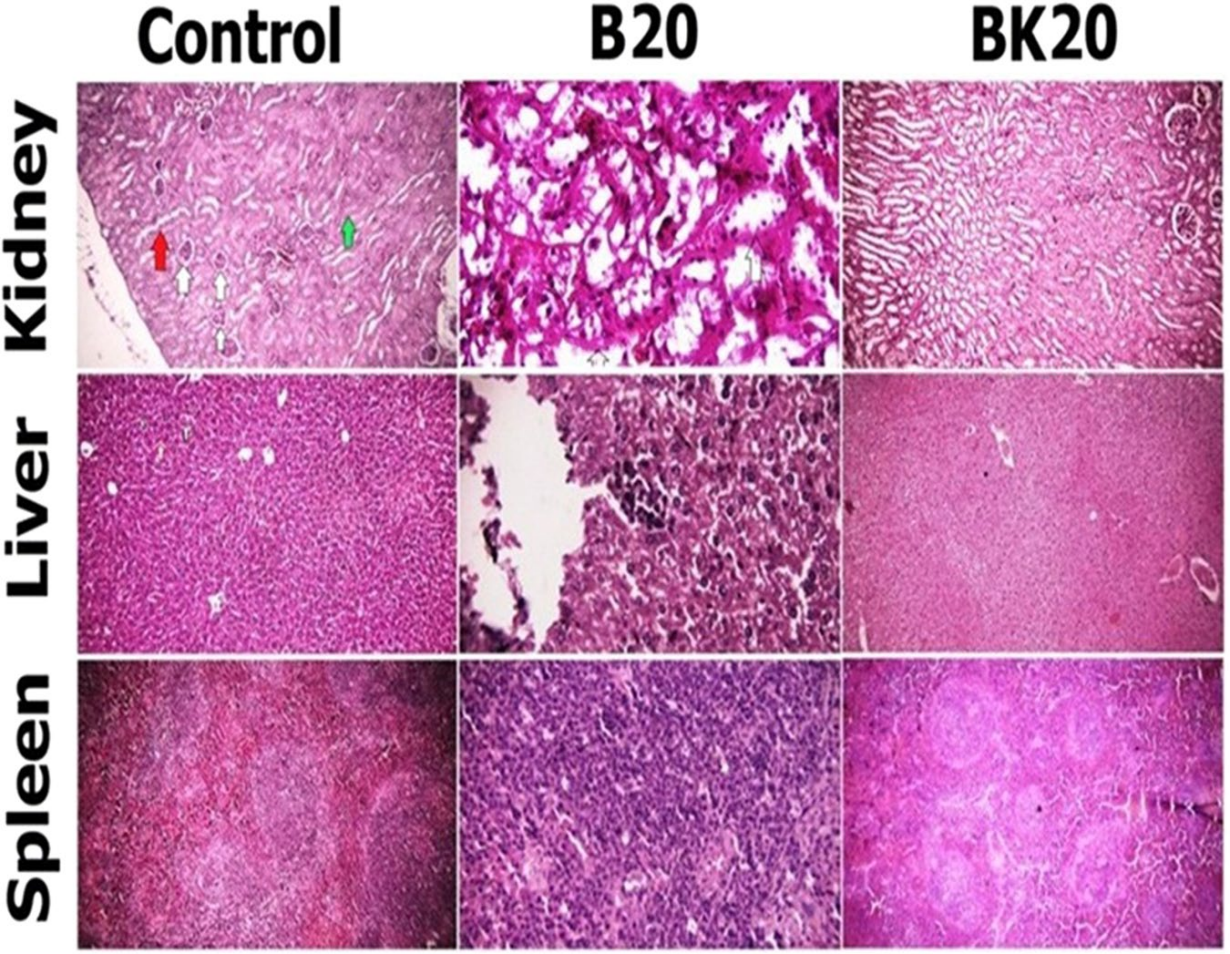
The results of histopathological toxicity between B20 mg/kg and nanodrug BK20 mg/kg. Histopathological toxicity was observed in B20 mg/kg receiver mice. The toxicity effects included hepatocytes degeneration (liver); pyknosis in kidney and unclear of border zone between white and red pulps in kidney; and hyperemia in spleen, while there was no toxicity in BK20 mg/kg receiver mice.

The aim of this experiment was to determine the non-toxic dose and for this purpose seven groups of healthy Balb/c mice were used. Non-treated healthy mice as negative control group and healthy mice received the solvent (a modified solvent of 10% acetic acid with appropriate pH for administration) was regarded as vehicle control. Also, Glucantime 200 mg/kg (GUL200 mg/kg) receiver mice were considered as positive control group. The other four groups were B10, BK15, 20 and 20 mg/kg. This arrange of groups was regarded throughout the *in vivo* toxicity evaluation including evaluation of enzymatic toxicity, mortality rate and pathological effects (Fig. 14).

The results showed that the serum concentrations of creatinine, Blood Urea Nitrogen (BUN), Aspartate Transaminase (AST), Alanine Transaminase (ALT) and Alkaline Phosphatase (ALP) in nanodrugs BK10, 15 and 20 mg/kg receiver mice were normal, while these values inB20 mg/kg receiver mice were significantly increased compared to control group (P ≤ 0.001) (supplementary file). Therefore, the highest dose of BK as BK20 mg/kg was selected for treatment. It should be noted that the results of enzymatic toxicity of main formulations were mentioned and the results of non-toxic formulations were not mentioned.

The mortality rate was 20% in B20 mg/kg receiver mice while all of the nanodrug BK20 mg/kg receiver mice were alive at the end of the study.

In addition, the results of histopathological toxicity showed morphological changes in liver, kidney and spleen in B20 mg/kg receiver mice while these tissues were normal in nanodrug BK20 mg/kg group. In B20 mg/kg receiver mice, the degenerative effects were observed in liver cells, whereas hyperemia and pyknosis were distinguished in the kidney tissue. In addition, in B20 mg/kg group border zone of red and white pulps in spleen of mice were not determined while all of the evaluated tissues were normal in nanodrug BK20 mg/kg receiver mice. Therefore, the highest dose of the nanodrug which was BK20 mg/kg was selected for evaluation the therapeutic effects (Fig. 14). It should be noted, in Fig. 14, the pathology results of those formulations were mentioned which caused tissue toxicity and pathology results of non-toxic formulations (vehicle, GUL200, BK10, BK15 mg/kg) were not shown.

### Nanoformulations efficacy on the lesion size

**Figure 15:**
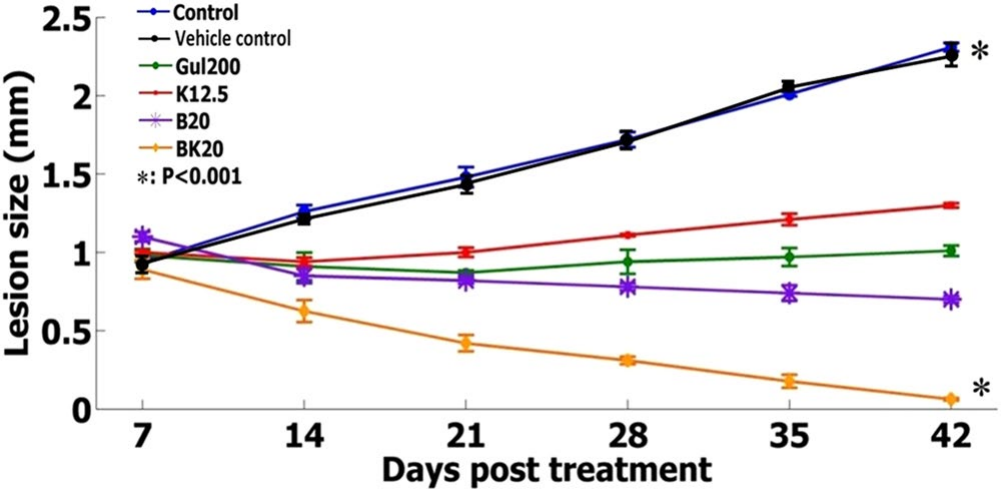
The lesion size in L. major-infected mice received various formulations. As the figure shows, the lesion size reached to zero in nanodrug BK20 mg/kg receivers compared to negative control group (non treated infected Balb/c mice) and GUL receiver as positive control (P < 0.001).

The formulations efficacy was evaluated on the following six groups including 1: negative control group (non-treated L. major infected Balb/c mice), 2: positive control group (GUL200 mg/kg), 3: vehicle control (modified solvent of 10% acetic acid with appropriate pH), 4: B20 mg/kg, 5: BK20 mg/kg and 6: K12.5 mg/kg. The results showed that the lesion size was biggest equal to 2.31 mm in negative control groups (non-treated L. major infected Balb/c mice and vehicle control group). The lesion size in positive control group (GUL200) was negligibly decreased to 1.2 mm. Also, in B20 mg/kg and K12.5 mg/kg receiver mice, the lesion size was slightly decreased, while in the group of BK20 mg/kg the lesion size was considerably decreased and reached to zero (P < 0.001) (Fig. 15).

### The pathological findings of infected footpad in mice of different groups

The pathological findings were evaluated on the following seven groups including 1: negative control group (non-treated L. major infected Balb/c mice), 2: positive control group (GUL200 mg/kg), 3: vehicle control (modified solvent of 10% acetic acid with appropriate pH), 4: B20 mg/kg, 5: BK20 mg/kg, 6: K12.5 mg/kg and 7: healthy mice.

#### Healthy group (Balb/c mice)

The results showed that foot skin has normal structure such as a stratified squamous epithelium, intact peg and papilla, dens connective tissue in dermis in palmar side of foot. Hair follicles and sebaceous glands were also seen at dorsal side of foot skin. Between the two sides of foot, skeletal muscles and bone could be seen with no involvement.

#### Negative control group (Infected non treated group)

**Figure 16:**
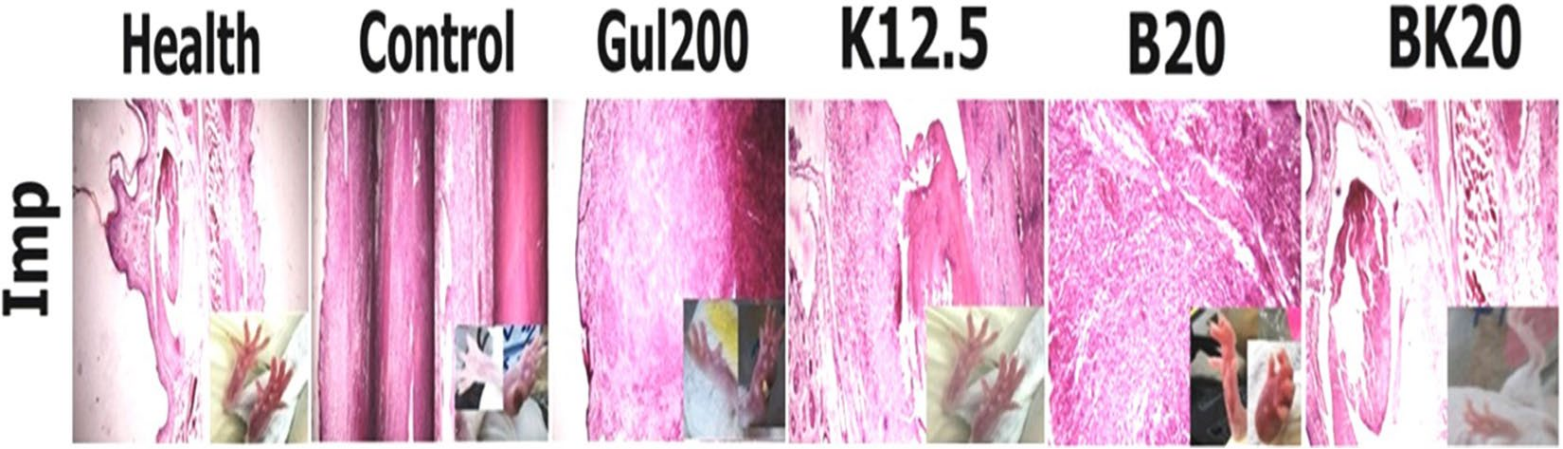
*In vivo* histopathological effects of H&E staining in L. major-infected footpad of Balb/c mice treated with four formulations of Gul200 mg/kg (positive control group), K12.5 mg/kg, B20 mg/kg, BK20 mg/kg and negative control group (non-treated infected mice). The results showed that infected footpad in BK20 mg/mg receiver mice was perfectly improved compared to negative and positive control groups.

Loss of skin epithelium, granuloma inflammation, hyperkeratosis, dermatitis and ulcer were the lesions which seen (Fig. 16).

#### Negative control group (vehicle control)

Loss of skin epithelium, granuloma inflammation, hyperkeratosis, dermatitis and ulcer were the lesions which seen. Therefore the findings were similar to negative control group (Fig. 16).

#### Positive control group (GUL200 mg/kg group)

A cloudy degeneration was seen in keratinocytes inflammatory cells infiltration beneath the wound necrotic tissue extended to metatarsal bone. Small granulomatous inflammation was observed in dermis (Fig. 16).

#### K12.5 mg/kg group

Footpad skin epidermis was normal. Granulomatous inflammation in dermis, extended into metatarsal bone (Fig. 16).

#### B20 mg/kg group

Normal stratified squamous epithelium and dense connective tissue were seen. However, inflammatory cell infiltration especially macrophages was exist which form a granulomatous inflammation (Fig. 16).

#### BK20 mg/kg group

Normal skin tissue was seen in palmar side of the footpad including stratified squamous epithelium, dense connective tissue. The footpad was completely found healthylike the footpad in healthy group (Fig. 16).

### Parasite burden measurement by Limiting Dilution Assay (LDA) method

The formulations efficacy was evaluated on the following six groups including 1: negative control group (non-treated L. major infected Balb/c mice), 2: positive control group (GUL200 mg/kg), 3: vehicle control (modified solvent of 10% acetic acid with appropriate pH), 4: B20 mg/kg, 5: BK20 mg/kg and 6: K12.5 mg/kg.

**Figure 17:**
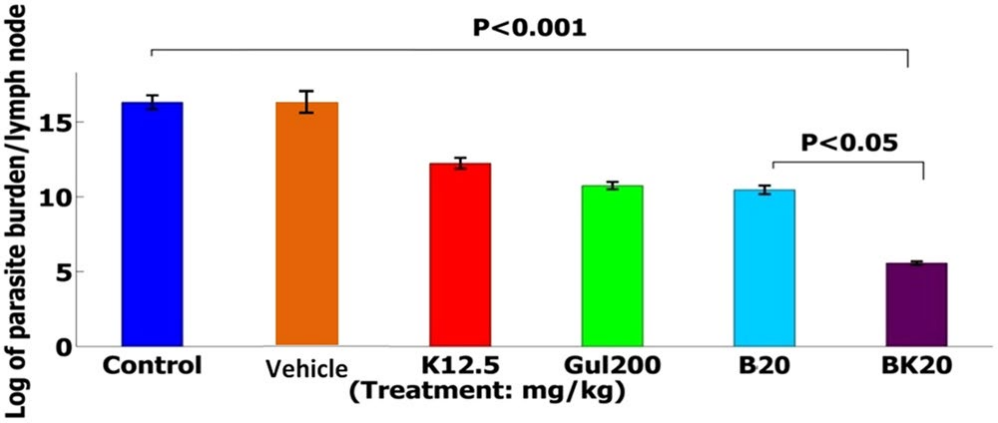
The evaluation results of parasite burden showed that BK10 mg/kg (8 × 10^6^) decreased the parasite burden to a large extent compared to negative control (non treated infected Balb/c mice) (16 × 10^6^) and GUL receiver as positive control (P ˂ 0.001) using LDA assay. The results are expressed as Mean ± SD.

The results showed that the highest parasite burden was measured in negative control group (non-treated and vehicle group) equal to the log of 16.32 × 10^6^. Also, in positive control group (GUL200), the parasite number was slightly decreased equal to the log of 10.75 × 10^6^. Further, B20 mg/kg with the log of 10.47 × 10^6^ and K12.5 mg/kg with the log of 11.35 × 10^6^ could slightly inhibit the parasite, while BK20 mg/kg significantly decreased the parasite burden with the log of 4.57 × 10^6^ compared to negative and positive control groups (P ˂ 0.001) (Fig. 17). Therefore, BK20 mg/kg was considerably effective in parasite inhibition.

### Histopathological results of Parasite number in different groups received various formulations by using Giemsa staining in infected liver, spleen and kidney and Footpad

The formulations efficacy was evaluated on the following six groups including 1: negative control group (non-treated L. major infected Balb/c mice), 2: positive control group (GUL200 mg/kg), 3: vehicle control (modified solvent of 10% acetic acid with appropriate pH), 4: B20 mg/kg, 5: BK20 mg/kg and 6: K12.5 mg/kg.

The tissues of liver, spleen, kidney and infected are selected based on Belosevic *et al*. study^23^. They used of Giemsa staining method for histopathological evaluation. Amastigotes of the parasites in all Hematoxylin and Eosin (H&E) stained tissues (supplementary file) were detected and confirmed by giemsa staining. In the present study, the observations in various tissues were as follow:

#### Liver

**Figure 18:**
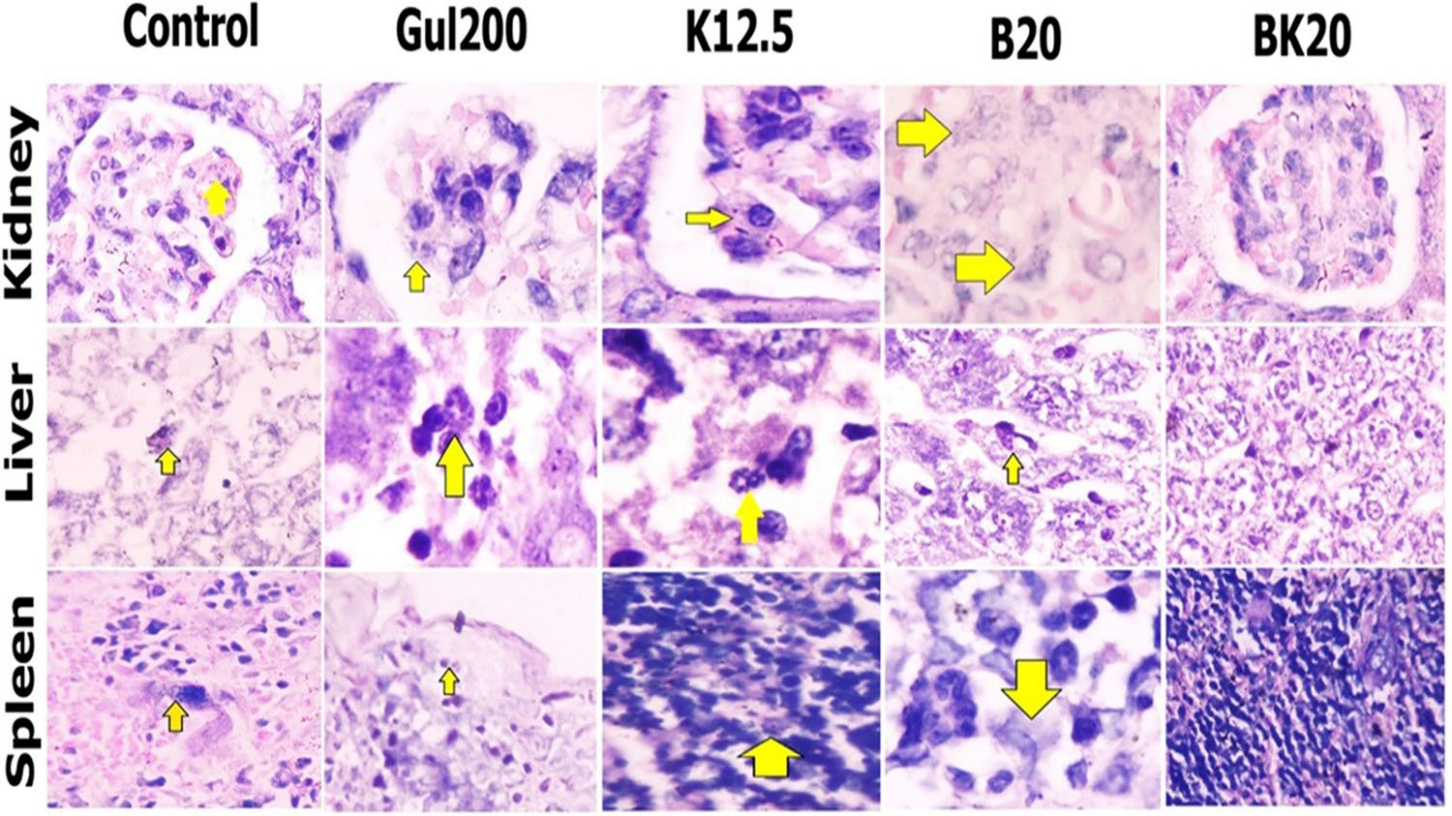
*In vivo* histopathological evaluation through giemsa staining for measurement of parasite number in kidney, liver and spleen and footpad in different mouse groups received Gul200 mg/kg (positive control group), K12.5 mg/kg, B20 mg/kg and BK20 mg/kg compared to the negative controlgroup (non-treated infected mice). The results showed that there was no parasite in BK20 mg/kg receiver mice compared to positive and negative control groups.

The parasite number in kupfer macrophages in negative control group (non-treated infected mice and vehicle control group with the parasite number of 5) was high and in positive control group (GUL200) was less compared to the negative control group. Also, the parasite number was slightly decreased in B20 mg/kg and K12.5 mg/kg receiver mice compared to the negative control group. The results showed that there was no parasite in nanodrug BK20 mg/kg receiver mice, therefore, BK20 mg/kg was succeeded to decrease the parasite number (Table 1) (Fig. 18).

**Table 1:** Parasite number in different tissues of L. major-infected Balb/c mice evaluated by light microscopy in 10 fields of view observed by 40X objective magnification.

Groups	The number of tissue amastigotes				
	Footpad	Lymph node	Spleen	Kidney	Liver
PBS	20	10	3	3	5
GUL	4	4	2	0	3
B	5	4	2	0	3
K	6	6	2	0	3
BK	0	0	0	0	0

#### Spleen

The parasite number in spleen macrophages in negative control group (non-treated infected mice with the parasite number of 3) was high and in positive control group (GUL200) was less compared to the negative control group. Also, the parasite number was slightly decreased in B20 mg/kg and K12.5 mg/kg receiver mice compared to the negative control group. However, there was no parasite in nanodrug BK20 mg/kg receiver mice, therefore, BK20 mg/kg was succeeded to decrease the parasite number (Table 1) (Fig. 18).Totally, the parasite number in the spleen is less compared to the liver. This finding is reported by Ribeiro *et al*. study^14^.

#### Kidney

The penetration depth of the parasite was observed in the kidney of negative control group(non-treated infected mice and vehicle control group and there was no parasite in the kidney of other groups (Table 1) (Fig. 18).

#### Footpad

The parasite number in footpad macrophages in negative control group (non-treated infected mice and vehicle control group with the parasite number of 20) was high and in positive control group (GUL200) was less compared to the negative control group. Also, the parasite number was slightly decreased in B20 mg/kg and K12.5 mg/kg receiver mice compared to the negative control group. The results showed that there was no parasite in footpad of nanodrug BK20 mg/kg receiver mice (Fig. 2 in supplementary figures), therefore, BK20 mg/kg was succeeded to decrease the parasite number (Table 1).

## Discussion

In the present study, it was aimed to development a novel nanodrug and increase the effective therapeutic dose for leishmaniasis. In this regard, a novel solvent was designed to achieve the desired dose.

In contrast to other researchers which used of ionic gelation for synthesizing of K, the phase separation method was used here to synthesize the K. The particles were synthesized with the size of 102 nm and then B was loaded into nanoparticles by using phase separation method with the drug loading efficiency of 93% and the size of 124 nm for BK. The particles were physicochemically characterized and the drug loading into nanoparticles was approved. In this regard, the results of drug delivery and cellular uptake showed that the drug release pattern from nanoparticles is slow release with cellular uptake of 97.5%. The results of cellular uptake were then confirmed by fluorescent microscopy. Next, the potency of the formulations to produce the NO in macrophages was evaluated and measured. The results showed that BK20 and K12.5 mg/kg could increase the NO concentration in some extent. In the next step, due to requirement of the effective therapeutic dose (BK20 mg/kg), a novel solvent containing 10% acetic acid was used. The results showed that nanodrug BK20 mg/kg was completely non-toxic *in vitro* and *in vivo* environments. Therefore, the highest drug dose equal to 20 mg/kg was selected for treatment of L. major. The results of *in vitro* and *in vivo* showed that BK20 mg/kg was effective by 100% in the wound healing and to a large extent in parasite inhibition.

Initially, in various studies to dissolve the B’s solubility problems, K is synthesized^24–26^. There are various methods for K synthesis^27^. In this regard, various investigators have used of these methods for K synthesis. The size of K synthesized by various investigators was found to be 114 nm^14^. However, commercial K nanoparticles were used by Asthana *et al*., Gupta *et al*., Jain *et al*., and Singh *et al*. characterized by low drug loading efficiency^18,26,28,29^.

Also, in the present study, contrast to other studies, K was synthesized for the first time by using phase separation method to increase the B solubility (briefly: chitosan solution + acetic acid solution + TPP → chitosan nanoparticles) with the size of 102 nm, zeta potential of 14 mV and PDI of 0.2 obtained by Dynamic Light Scattering (DLS) which was confirmed by AFM, SEM and TEM methods and the conformity was found among the results. Two distinct properties of synthesized K in the present study was its low molecular weight and high viscosity, resulted in more effective drug delivery and wound healing compared to other studies. The particles were more symmetrical and homogenized compared to K prepared with other methods. Also, FTIR spectrum of particles was obtained. Synthesis of K by using phase separation method is a cost effective, simple and time-saving method and leads to nanoparticles preserve their nano properties^30^.

After K synthesis, B was loaded into K by using phase separation method. Different methods of drug loading in micro/nanoparticulate systems are known as method a included incorporating the drug during the preparation of the particles (8% w/w of drug loading) and method b after the formation of the particles by incubation the drug with them (69% of drug loading)^31,2017]^. As far as the authors know, loading of B into K was not reported by now.

In the present study, B was loaded into K by using phase separation method for the first time. The drug loading efficiency of 93% was obtained by using standard curve (method b). Using method b was resulted in to have a high drug loading efficiency. The results showed that using phase separation method resulted in increasing of drug loading efficiency which in turn results in high drug solubility. The higher drug loading efficiency, the more effective drug delivery is achieved. In the all studies, the particles were characterized with different methods.

In the present study, the size of BK was 124 nm, PDI, 0.3 and zeta potential was 8 mV. Increasing of PDI from 0.2 in K to 0.3 in BK indicated development of a new aggregation probably due to interaction between the drug and nanoparticles^32^. Therefore, this aggregation caused the PDI to be increased.

In the present study, prepared nanodrug BK was characterized by DLS and its results were confirmed by SEM, AFM and TEM methods which were in accordance to each other. The fiber-like structure is observed in AFM images while in TEM microscopy, the spherical particles were formed. This contradictory resulted from difference in sample preparation for TEM and AFM imaging, in which in TEM imaging the samples were severely purified and sonicated to increase the dispersity, while these processes were not performed for AFM imaging. In AFM imaging, a thin layer of concentrated sample was prepared and then imaging was performed. The TEM figures prepared in the present study have more conformity with the TEM figures in literature in that the particles were formed spherical^33^. The TEM figures were more accurate in the present study due to the process of sample preparation.

Also, SEM characterization showed synthesizing of symmetrical BK nanoparticles. Further, physical drug loading was confirmed by FTIR and H-NMR techniques as well and the FTIR results were confirmed by H-NMR method. Moreover, SEM provides larger figures from the molecule and shows the morphology of particles at the molecular scale, therefore molecular morphology is better diagnosed by SEM compared to AFM. However, AFM assesses the particles at the atomic level and provided more details of the atomic morphology and provides atomic figures of the particles. Further, AFM provides two and three dimensional figures of the particles. Totally, we used of SEM and AFM methods in order to detect and diagnose as well as analyze the particles more accurately and precisely. Also, it should be noted that both methods confirmed each other. The purity of K and BK was indicated by TLC chromatography. In addition, the results of XPS analysis indicated loading of B into K. After performing characterization, based on the protocols available in the literature, the drug delivery system analysis including drug release and cellular uptake were performed. Initially, drug release study was performed. There are two modes of drug release including *conventional*^25^ with a burst release and the whole of drug is released, therefore booster dose is needed^34^ and *controlled release* which is a slow drug release and the booster dose is not required^34^. Slow drug release from carriers can reduce the drug toxicity^35^ and gives nanoformulations with prolonged release profile^36^. Drug release from nanoparticles determines the drug therapeutic effects^37^. This property of chitosan (slow drug release) decreased the drug toxicity. Mechanisms by which K functions as slow drug release carrier are included (a): drug release from the surface of particles, (b): diffusion through the swollen rubbery matrix, (c): release due to surface erosion^38^, d): the presence of TPP in chitosan nanoparticles along with these three mechanisms and (e): acid resistive property^17,24,26^. Acid resistive property of K makes the nanoparticles to overcome immediate lysosomal digestion in macrophages, therefore chitosan targets the sites of reticuloendothelial system (RES) organs which are the host for intracellular leishmania parasites^17^.

The results of present study showed that, 30% of loaded drug was released in the first six hour of the study and 90% of total loaded drug was released after 48 h. Therefore, the pattern of drug release was slow release. It was resulted from the presence of TPP in the structure of K synthesized by phase separation method. Slow drug release provides more prolonged of drug exhibition into the target cells such as macrophages which are the host for intracellular leishmania parasites^17^. In the next step, second analysis of drug delivery system was evaluating the cellular uptake.

Researchers evaluated the drug penetration rate by using cellular uptake assay. The results of a study showed that chitosan coated nanoparticles had two times cellular uptake compared to the uncoated nanoparticles in both L. donovani-uninfected and infected J774A.1 cells^18^ due to surface positive charge of K resulted in more rapidly and more taken up by macrophages and other phagocytic cells and immediately opsonisation after application due to the high molecular weight, therefore high cellular uptake of chitosan is occurred in macrophages^17,24^.

The results of the present study showed that B’s cellular uptake was increased significantly after loading of the drug into K and this increase is resulted from the positive surface charge of K and high drug loading efficiency which resulted in enhancement of opsonisation and phagocytosis. The results of our study showed the nanodrug cellular uptake of 97.5% which indicated by flow cytometry method and confirmed fluorescent microscopy.

Investigators in the next step, evaluate the drug effects and determine the non toxic dose of a produced drug *in vitro* environment. They, firstly evaluate the formulation toxicity in cell culture by using MTT assay to determine the administered dose and toxicity. The results of various studies showed that K is used as drug carrier in various studies and the results showed that K is able to decrease the cell toxicity^18,25,26,29^. In addition, it has been proved that K is non-toxic and FDA approved^38^.

In the present study, the negative control group was peritoneal macrophages and positive control group was A20 µg/ml group and the results showed that nanodrug BK decreases the B toxicity by 100%. This decreasing of B toxicity is due to coverage of the drug by K and reduction of drug toxicity by this nanocarrier. The external coverage of nanodrug is in contact with the cells resulting in decrease the drug toxicity. In addition, after nanodrug transferring into the cells and slow drug release, the drug was slowly exhibited to the cells and as a result the drug toxicity was decreased. Totally, due to K properties including reduction of drug toxicity and slow drug release, the drug toxicity is decreased by 100%.

In the next step, investigators have evaluated the therapeutic effects of nanoformulations on the parasite promastigotes *in vitro* environment.

In this regard, Chowdhury *et al*. have shown the growth inhibitory effects of B on promastigotes of L. donovani^12^. Also, Sousa *et al*. showed that 50 µg/ml of B could kill 50% of L. infantum promastigotes^5^. Therefore, these researchers could achieve a low killing effects of B on parasite promastigote, while in the present study compared to previous study, BK20 µg/ml at the lower dose showed the higher killing effects of 86% against promastigotes of L. major. This enhanced killing effects of BK results from the killing effects of K and slow drug release from K.

It should be noted that in this test, reducing the parasite viability indicated the enhancement of the parasite killing effects which and as in turn indicated the effective killing effects of nanodrug on the promastigote of parasite. In this test, negative control group was L. major promastigotes and positive control group was A20 µg/ml. The solvent used for evaluation the killing effects of formulations was PBS.

In the next step, the therapeutic effects of nanodrugs were evaluated against the amastigote forms of Leishmania in various studies. The antileishmanial activity of B has been evaluated against amastigote forms of L. donovani^9^. In this regard, Alakurtti *et al*. showed that B at the dose of 25 µg/ml, killed 75% of the parasite^9^.

While, in the present study compared to the previous study, BK20 µg/ml increased the inhibition effects by 81% against amastigote forms of L. major compared to the negative control group at the lower dose. In this test, increasing the inhibition effects of nanodrug indicated its enhanced killing effects, therefore the amastigote viability was reduced. Thus, the more antiparisitic effects of nanodrug, the more killing effects against parasite was achieved.

This enhanced antiparisitic activity results from the antileishmanial effects of chitosan, the synergistic antileishmanial effects of B and K, slow drug release of nanodrug BK, positive charge of nanodrug BK, high cellular uptake of nanodrug BK, and immunologic effects of K.

In the next step, the potency of formulations to produce NO in macrophages was evaluated. The obtained results were statistically analyzed. In this test, the various groups were LPS as positive control, healthy macrophages normal group (negative control) and L. major infected macrophages as negative control group. The results showed that BK20 µg/ml and K10 µg/ml increase the NO concentration to some extend compared to other groups indicating the K effects on macrophage activation. The functional mechanism of K through phagocytosis or endocytosis in macrophages leads to induction of proinfalmatory cytokines and activation of the macrophages, resulting in enhancement of NO concentration. Totally, our results showed that the BK was succeeded in parasite inhibition due to increasing the effective dose of B (B functions by apoptosis induction) and K functional mechanism. Therefore, these two reasons led to BK was considerably effective in parasite killing.

Totally, the results of *in vitro* evaluation proved that K as a targeted drug delivery system and antileishmanial agent could be considered for the disease treatment and nanodrug BK was effective to killing the promastigotes and inhibit the amastigote forms of the parasite.

In the next step, researchers evaluated the *in vitro* results of therapeutic effects *in vivo* environment. The results of literature review showed that the highest dose of B used for treatment of leishmaniasis was 10 mg/kg which was not considerably effective in the treatment. Using low dose of B was due to its low water solubility. Therefore, in the present study to solve this problem, in addition to use of K, prepare a novel solvent was considered to increase the effective dose. Therefore, a panel of various solvents (salt bile, tween 80, DMSO, ethanol, methanol and acetic acid) was prepared and the highest dose of solubility was achieved with novel solvent (10% modified acetic acid with modified pH). This solvent was able to increase the B solubility by 300 folds and BK solubility of 20 mg/kg was achieved. Therefore, using phase separation and designed solvent increased the bioavailability and efficacy of the drug as well as reducing its side effects. After development of the nanodrug as a proper dose for treatment, researchers evaluate the blood factors to determine the administered dose and toxicity evaluation.

To evaluate the toxicity effects *in vivo* environment, based on scientific protocols researchers always use of healthy mice. The results of various studies showed that enzymatic toxicity of drug was reduced after loading into K^14,18,26^. To *in vivo* toxicity evaluation, 7 groups of healthy female Balb/c mice (n = 10) were selected as group 1: negative control group (non-treated healthy Balb/c mice), 2: positive control group (GUL200 mg/kg), 3: vehicle control (modified solvent of 10% acetic acid with appropriate pH), 4: B20 mg/kg, 5: BK10 mg/kg, 6: BK15 mg/kg and 7: BK20 mg/kg.

In the present study, the results showed no toxicity in mice received nanodrug BK10, 15 and 20 mg/kg compared to B20 mg/kg which was toxic. Therefore, the highest dose of BK20 mg/kg was considered as the therapeutic dose.

Researchers measured the mortality rate in the drug receiver healthy mice to evaluate the drug toxicity^18,26^. The results of various studies showed that K decreased the mortality rate of drug^29^.

In the present study, the mortality rate was measured in nanodrug BK20 mg/kg and B20 mg/kg groups of mice and the results showed that 20% of mice received B10 mg/kg were dead whereas all mice in nanodrug BK20 mg/kg group was remained alive at the end of study due to antitoxic effects of K^38^.

In the next step, researchers evaluated the drug effects on different tissues of mice in order to evaluate the drug toxicity effects^14,18,26^. The toxicity results of histopathological studies in different studies showed that the K decreased the tissue toxicity of drug^14,18,26,29^.

In the present study, the toxicity of nanodrug was investigated on liver, kidney and spleen tissues by H&E staining method. The results of present study showed the toxicity effects in kidney, liver and spleen of mice which received B20 mg/kg, whereas these effects were not observed in nanodrug BK10 mg/kg group. Therefore, this toxicity reduction is resulted from the potency of K in decrease the drug toxicity, slow drug release from K as well as safety of designed solvent. Therefore, the highest dose of BK as BK20 mg/kg was considered as non-toxic dose for evaluating its therapeutic efficacy.

To evaluate the therapeutic effects *in vivo* environment based on scientific protocols, researchers always use of infected mice. In the next step, the drug therapeutic effects were evaluated by measurement of lesion size in leishmania infected footpad of mice^14,17,18,26,29^. The results of previous studies showed that K decreased the lesion size by its own in leishmaniasis^14^.

In the present study, the therapeutic effects of formulations were evaluated on six groups of mice including 1: negative control group (non-treated L. major infected Balb/c mice), 2: positive control group (GUL200 mg/kg), 3: vehicle control (modified solvent of 10% acetic acid with appropriate pH), 4: B20 mg/kg, 5: BK20 mg/kg and 6: K12.5 mg/kg. The formulations were administered intraperitoneally for 6 weeks and alternative day. The results of various studies showed that the drug toxicity decreased in intraperitoneal injection compared intravenous^14^.

In the present study, the results showed that BK20 mg/kg reduced the lesion size to zero due to antileishmanial effects of K, synergistic antileishmanial effects between B and K, immunologic effects of K and the K effects in wound healing.

In various studies, researchers evaluated the pathological effects of infected footpad in order to confirm lesion size measurement^39–42^. The results of a study showed that the more successful the treatment, the less pathological lesions have seen^43^.

The results of literature review showed that chitosan causes wound healing due to enhancing vascularization and the supply of chito-oligomers at the lesion site, which have been implicated in better collagen fibril incorporation into the extracellular matrix, deliver fibroblast growth factor-2 (FGF-2) and viscosity^38^. Also, K is able to stimulate Th1 immune responses and as a result improved the efficacy of loaded drug on leishmaniasis lesions^14^.

In the present study, histopathological studies of L. major was performed and the results showed that BK20 mg/kg caused wound healing by 100% compared to positive control group (GUL200 mg/kg) as well as negative control group (infected non-treated group). Therefore, the reasons of success of BK20 mg/kg are the ability of chitosan to increase vascularization and the supply of chito-oligomers at the lesion site resulting in wound healing, high viscosity of nanodrug BK20 mg/kg, cellular uptake of more than 96% and slow-controlled drug release. Therefore, a conformity was observed between the results of lesion size and pathological findings.

Also, parasite burden was measured in previous studies to evaluate the drug effects and the results of lesion size were confirmed by measurement of parasite burden^12^. Chowdhury *et al*. used of the B at the dose of 10 mg/kg in the leishmaniasis and the results showed that the parasite burden was decreased negligibly^12^, while the results of present study showed that BK20 mg/kg significantly decreased the parasite burden. This reduction of parasite burden by BK20 mg/kg is resulted from increasing the administered dose.

Based on the previous studies^39–42^, to confirm the LDA results, parasite number was measured in different tissues. To evaluate the therapeutic effects of nanoformulations, the parasite number in footpad, liver and spleen was enumerated by using H & E and giemsa staining methods^39–42^. The parasite killing effects of K on liver and spleen were evaluated in a study and the results showed that these effects were more potent in spleen due to higher numbers of Th1 cells^14^ and due to targeted delivery of chitosan into macrophages, K concentration is increased in macrophage rich organs such as liver and spleen and macrophages. From the other hand, macrophages are the host of leishmania parasite. Therefore, these organs (liver and spleen) were chosen for evaluating the parasite burden^17^.

The results of present study showed that, parasite number was reached to zero in different tissues in BK20 mg/kg receiver mice after evaluating 10 fields under microscope, whereas in non-treated infected mice (negative control group), there are many parasites in these tissues. Also, the negligible parasite number was found in GUL200 mg/kg receiver mice. Therefore, BK20 mg/kg could completely succeeded in the treatment of L. major due to increase of the used dose.

On the other hand, based on literature review, the renal presence of the parasite has not been reported by now. Interestingly, the parasite was found and counted in the kidney tissue of negative control group. However, there was no parasite in the kidney tissue of treatment groups.

The results of present study showed that BK20 mg/kg along with novel solvent were succeeded to decrease the parasite burden, lesion size, parasite number and pathological effects and the *in vitro* and *in vivo* results were in accordance and the results of parasite burden, lesion size, parasite number and pathological effects showed that BK20 mg/kg was succeeded in the treatment of L. major by 100%, in which compared to positive control group of GUL200 which was negligibly effective to reduce the parasite burden and lesion size, BK20 mg/kg was completely succeeded to wound healing by 100%, this successfully is results from increasing in the used dose of B (B induce apoptosis in the parasite), antileishmanial effects of K, slow drug release from K, high cellular uptake and high drug loading efficiency. Therefore, BK20 mg/kg can be considered as a proper alternative regimen to in the treatment of L. major.

## Conclusion

Overall, due to chitosan synthesis by using phase separation and drug loading by phase separation, novel solvent (modified 10% acetic acid solvent) and enhancing the therapeutic dose of BK to 20 mg/kg, the successfully treatment of L. major was achieved *in vitro* and *in vivo* environments in terms of improvement the treatment indicators. BK20 mg/kg was non-toxic by 100% *in vitro* and *in vivo* environments. Therefore, BK20 mg/kg can be a proper alternative for leishmania treatment.

## Suggestions

Considering the proper characteristics of BK, it could be considered as a novel therapeutic agent in the treatment of leishmaniasis with the ability to overcome the drug resistance. Since, B is a low cost herbal derived compound, therefore could be as proper alternative for current leishmaniasis treatment regimens.

## Materials and Methods

### Materials

B was purchased from Baoji Guokang Bio-Technology Co.,Ltd (China). Chitosan (20 KDa), 3-(4,5-Dimethylthiazol-2-yl)-2,5-diphenyltetrazolium bromide) (MTT) and acetic acid were obtained from Merck company (Germany). Also, culture medium RPMI-1640, Fetal Bovine Serum (FBS) and Penicillin/Streptomycin antibiotics were supplied by Gibco Company (USA). Furthermore, Tripolyphosphate (TPP), DMSO and Schneider culture medium were prepared form Sigma-Aldrich (USA). Iranian strain of L. major (MRHO/IR/75/ER) was supplied by Pasteur Institute of Iran. All other materials were of analytical grade. Double distilled water was used throughout the study.

### Methods

#### Preparation of K

K was prepared by phase separation method based on literature with slightly modifications^38,44^. In this method, electrostatic interactions between chitosan with positive charge and TPP with negative charge are determinant factors for forming the nanoparticles. Briefly, chitosan solution (10 ml, 1 mg/ml) was prepared in 0.05% acetic acid solution and stirred for 5 minutes. Then, solution of TPP (3 ml, 0.25% w/v) was added and stirred for 5 min (300 rpm) again. Next, the nanoformulation was dialyzed by using a 6 KDa cut off dialysis bag against PBS buffer to remove any impurity and the suspension of nanoparticles was washed three times by using ultracentrifuge (13000 rpm, 4 °C, 30 min). Next, the prepared nanoparticles were stored in 2 ml vials in order to dehydration and purification by using liophilization.

#### Preparation of nanodrug BK

Nanodrug BK were prepared by using drug adsorption and phase separation methods. For this purpose, 8.5 ml of PBS was added to the nanoparticle precipitate prepared in previous section and stirred (300 rpm). Then, 1.5 ml of drug solution (20 mg/ml in DMSO) was added to the suspension and stirred for 7 days (300 rpm). Next, the nanoformulation was dialyzed against PBS buffer by using a dialysis bag (molecular weight cut off = 6000 Da) to remove any impurity and the suspension was ultracentrifuged three times with PBS (13000 rpm, 4 °C, 30 min)^38,44^. The ratio of B and K in BK formulation was 20:12.5 mg. Also, the purity of K and BK formulations were evaluated by TLC and XPS analysis.

#### Determine the drug loading efficiency in nanodrug BK

Firstly, the curve of B was prepared. For this purpose, supernatant obtained from drug-loaded nanoparticles was diluted and their absorbance was read at 405, 409 nm using spectrophotometer for five times. After that, the drug concentration in supernatant was determined by using free curve. Then, drug loading efficiency was calculated by using formula below^17,26^:

$${\rm{Drug}}\,{\rm{loading}}\,{\rm{efficiency}}\,( \% )=\frac{{\rm{Amount}}\,{\rm{of}}\,{\rm{initial}}\,{\rm{drug}}\,({\rm{mg}})-{\rm{amount}}\,{\rm{of}}\,{\rm{drug}}\,{\rm{in}}\,{\rm{supernatant}}\,({\rm{mg}})}{{\rm{Amount}}\,{\rm{of}}\,{\rm{initial}}\,{\rm{drug}}\,({\rm{mg}})}\times 100$$

#### Determine the size, size distribution and zeta potential

Size, size distribution and zeta potential of K and nanodrug BK were determined by using DLS method and Zetasizer instrument (Malvern Instruments Ltd., Malvern, Worcestershire, UK). For this purpose, nanoparticle suspension was diluted and the absorbance was read at 630 nm and then was introduced to the instrument^17,26^.

#### TLC

The samples of K, B and BK (2 µl) were mounted on a silica plate and left to dry at room temperature. Afterthat, the plate was placed at 45 °C angle in a chamber containing 10% methanol in chloroform. After 45 min the plate was exited and allowed to dry. The components were detected by using visualizing agent, iodine vapours and their related R_f_ were obtained.

#### XPS analysis

The powder of K and BK formulations (2 mg) were placed on a layer of silicon and the XPS spectra were obtained using a specific Phi XPS system at the basic pressure of 10 × 10^-2^ torr. X-rays were obtained by using a monochromatic quartz crystal A1K. The measurements were carried out by using a transmission energy of 47 eV (with resolution of 0.64 eV). An angle electron collection of 45 °C was considered for all measurements at normal level.

#### Evaluation the K and BK morphology by using SEM

For this purpose, a drop of nanoparticles suspension was dried at room temperature and the related powders for each formulations were obtained. The powders were initially coated with a thin layer of gold and then it was evaluated with SEM instrument (SEM, MIRA II TESCAN) at 10 KV accelerating voltage^17,26^.

#### Evaluation the K and nanodrug BK morphology by using TEM

Suspensions of K and BK nanoparticles were firstly sonicated and dispersed. Then a drop of each one was mixed on a carbon coated copper grid and dried at room temperature. Next the samples were evaluated by using TEM instrument^17,26^.

#### Evaluation the K and nanodrug BK morphology by using AFM

The morphology of nanoparticles was also evaluated by using AFM (Nano Wizard II AFM, JPK Instruments, Berlin, Germany) method. Briefly, a drop of nanoparticles suspensions was placed on a mica surface and was dried at room temperature. The imaging was performed using tapping-mode in air and room temperature on a silicon cantilever with spring constant of 40 Nm^-1^. The obtained figures from nanoparticles were processed by using JPK software (Germany)^17,26^.

#### Evaluation the K and BK by using FTIR

Chemical structure of the loaded drug and the type of drug loading were determined by using FTIR method. For this purpose, K and nanodrug BK suspensions were centrifuged (13000 rpm, 30 min) and the related nanoparticles precipitates for each formulations were obtained. The precipitates were then dried at room temperature and mixed individually with potassium bromide. Next, the mixtures were individually pressed and related pellets for each formulations of K and BK were obtained which were evaluated by using FTIR instrument (Nicolet 740SX)^17,26^.

#### Evaluation the K and BK by using H-NMR

Structure, molecular properties and chemical bonds of nanodrug BK were evaluated by using H-NMR method. Briefly, 1 mg of nanodrug BK was dissolved in deuterium DMSO and then was evaluated by using H-NMR spectroscopy at FT pulse-mode at 300 MHz^17,26^.

#### The kinetic of drug release

The drug release study was performed by using dialysis membrane (molecular cut off 10 KDa) and standard curve. For this purpose, the suspension of nanoparticles was centrifuged (13000 rpm, 30 min) and the precipitate was obtained. Ten milligram of the precipitate was resuspended in 5 ml PBS and was poured in dialysis bag. Both sides of the bag were tightly closed and then immersed into graduated cylinders containing 100 ml PBS buffer. The cylinder was then placed on a stirrer and stirred (150 rpm). In the predetermined time intervals, 2 ml of buffer were withdrawn and replaced with 2 ml fresh buffer. The drug amount in isolated buffers was determined by using spectrophotometery method and the cumulative release curve was plotted^45^.

#### Measurement the cellular uptake of nanodrug BK using flowcytometry

Fluorescent property of compounds is used for determine their cellular uptake. Herein, peritoneal macrophages were cultured and after 4 h were treated with nanodrug BK at the concentration of 20 µg/ml. After 4 h incubation, the medium was removed and the nanodrug BK cellular uptake was measured by using flow cytometry instrument^17,26^.

#### Evaluation the cellular uptake of formulations by using fluorescent microscopy

As the previous section, macrophages were cultured and treated with free B and BK. After 4 h, the cellular uptake of formulations was evaluated by fluorescent microscopy.

### Biological activity of nanoparticles

#### The viability effects of nanoformulations on peritoneal macrophages

Macrophages were obtained from peritoneal lavage of Balb/c mice by using cold RPMI-1640 medium. In this study, female Balb/c mice (20 g, 8 weeks) were used. The animals were kept under controlled conditions of light (12 hours light/dark cycle), temperature (25 ± 2 °C), and humidity (40–60%). They were housed in polypropylene cages with free access to food and water throughout the study. Wood husk was used as bedding material and was changed daily. The animals were allowed to acclimate for a period of one week. In the present study, all animal experiments were approved by the Animal Experimentation Ethics Committee of Pasteur Institute of Iran. The peritoneal macrophages were cultured in 96-well plate at the density of 10^4^ per well in a 5% CO2 incubator. The culture medium was RPMI-1640 supplemented with 10% FBS and 1% Penicillin/Streptomycin. After 24 h, the culture medium was replaced with the culture medium containing B, K and nanodrug BK. The drug concentration of B and nanodrug BK were 5, 10 and 20 µg/ml and K were used at concentration of 10, 100 and 200 µg/ml. After 24, 48 and 72 h, the culture medium was replaced with MTT solution (10 µl MTT solution + 90 µl complete medium) and incubated for three hours. Next, the MTT solution was removed and 100 µl of acidic iso propanol was added to each well and incubated for 15 min. The absorbance was then read at 450 nm measurement wavelength and 570 nm reference wavelength filter using microplate scanning spectrophotometer (ELISA reader; Organon Teknika, Boxtel, the Netherlands). The viability was calculated by using formula below^46,47^:

$${\rm{Viability}}\,( \% )=\frac{{\rm{The}}\,{\rm{absorbance}}\,{\rm{of}}\,{\rm{cells}}\,{\rm{treated}}\,{\rm{with}}\,\mathrm{drug}\,}{{\rm{The}}\,{\rm{absorbance}}\,{\rm{of}}\,{\rm{control}}}\times {\rm{100}}$$

#### Killing effects of the nanoformulations on the promastigote

Promastigotes were cultured in 96-well plate at the density of 1 × 10^4^/well and 26 °C. The culture medium was RPMI-1640 supplemented with 10% FBS and 1% Penicillin/Streptomycin. The parasites were incubated with B20 µg/ml, K10 µg/ml and nanodrug BK20 µg/ml in which the drug concentration for B and nanodrug BK was 20 µg/ml and the concentration for K was 20 µg/ml. The incubation time was 48 h. After these times, the cytotoxicity effects of the formulations were evaluated using hemocytometer method in which the number of alive promastigotes was counted^48^.

#### Inhibition effects of the nanoformulations on the amastigote

Peritoneal macrophages were infected with Iranian strain of L. major promastigotes according to the previous study with some modification^49^. The infected macrophages were then cultured on coverslips and were incubated with free B20 µg/ml, K10 µg/ml and nanodrug BK20 µg/ml for 48 h. The K concentration was 10 µg/ml, while the drug concentration of B and nanodrug BK was 20 µg/ml. After these times, coverslips were washed and the macrophages infection rate was determined by using giemsa staining in which the remaining number of amastigotes was counted^50^.

#### Nitric Oxide generation assay

The amount of Nitric Oxide (NO) accumulated in culture media was measured by Griess reaction (G2930, Promega, Madison, WI) as a colorimetric assay. For this purpose, 50 µl of Griess reagent (1% sulfanilamide/0.1% naphthylethylenediaminedihydrochloride/2.5% H3PO4) was added to 50 µl of each sample in a 96-well microplate and the absorbance was measured at 540 nm by Elisa reader (BioTek Instruments, VT, USA). In this test, uninfected macrophages and L. major infect macrophages were regarded as negative control group, while L. major infected macrophages treated with B was regarded as positive control group.

#### The solvent design

To treatment of L. major infected mice, it was needed to use of 20 mg/kg of B which was not reported yet in the literature. This dose is an effective dose of B for treatment, therefore to dissolve this dose, a panel of various solvents (DMSO (10% v/v), methanol (7% v/v), ethanol (9% v/v), bile salt (2% w/v), NaOH (1% w/v), tween 80 (1% v/v), acetic acid (0–100% v/v) mentioned in literature was used which finally resulted in designing an appropriate solvent for achievement of the desired solubility (20 mg/kg)^14,17,24–26,51^.

#### *In vivo* toxicity of the nanodrug BK

For this purpose, 6 groups of female Balb/c mice (n = 10) were selected. They received the formulations of BK10, 15, 20 mg/kg, B20 mg/kg and the solvent intraperitoneally for 6 weeks and alternative day. After this time, animals were anesthetized and the heart blood samples were obtained. Then, the serum concentrations of BUN, creatinine, AST, ALT, and ALP were measured spectrophotometrically to determine the non-toxic dose. In this experiment, to determine the non-toxic dose, seven groups of healthy Balb/c mice were used. Non-treated healthy mice as negative control group and healthy mice received the solvent (a modified solvent of 10% acetic acid with appropriate pH for administration) was regarded as vehicle control. Also, GUL200 mg/kg receiver mice were considered as positive control group. The other four groups were B20, BK10, 15 and 20 mg/kg. This arrange of groups was regarded throughout the *in vivo* toxicity evaluation including evaluation of enzymatic toxicity, mortality rate and pathological effects.

Mortality rate was also measured in all seven groups of mice. In addition, to evaluate the toxicity effects, pathological studies were performed in these six groups of mice. Briefly, animals were anaesthetized and sacrificed by cervical dislocation. Then the organs of liver, spleen and kidney were removed and immersed in 10% formalin and paraffinized. Paraffinized tissues were cut into 5 µm sections, stained with H&E and evaluated with light microscope in oil immersion magnification (100X objective) for toxicity evaluation^52^. It should be noted the protocol of drug administration was similar in each part of *in vivo* toxicity evaluation.

#### Nanoformulations efficacy on the lesion size

L. major-infected Balb/c mice were made by using amastigotes inoculated into mice footpad^53^. After lesion development (~2.5 mm), animals were randomly divided into 6 groups (n = 10) and the lesion size was measured using a caliper. The six groups of L. major infected female Balb/c mice (n = 10) were as group 1: negative control group (non-treated L. major infected Balb/c mice), 2: positive control group (GUL200 mg/kg), 3: vehicle control (modified solvent of 10% acetic acid with appropriate pH), 4: B20 mg/kg, 5: BK20 mg/kg and 6: K12.5 mg/kg. The mice received the formulations intraperitoneally and every other day. After the first injection, lesion size was measured weekly and the results were recorded.

#### The pathological findings of infected footpad in mice receiving different formulations

The infected footpad in 6 groups of L. major infected female Balb/c mice including negative control group (non-treated L. major infected Balb/c mice), 2: positive control group (GUL200 mg/kg), 3: vehicle control (modified solvent of 10% acetic acid with appropriate pH), 4: B20 mg/kg, 5: BK20 mg/kg and 6: K12.5 mg/kg was harvested, immersed into 10% formalin and paraffinized. Then, the paraffinized tissue were cut at 5 µm thick sections and evaluated histopathologically using (H&E) staining. Tissue injuries were included any cellular changes, inflammation, edema and tissue granulation. For this purpose, Panoramic Viewer (Software version: 1.15) digital microscope (3DHISTECH Ltd., Budapest, Hungary) was used with 40X objective magnification.

#### Parasite burden measurement by using LDA method in popliteal lymph node

One week after the final intraperitoneal injection of the formulations of B20 mg/kg, nanodrug BK20 mg/kg, GUL200, K (12.5 mg/kg), the animals were anesthetized and then were sacrificed by decapitation. The groups of animals were as follow: negative control group (non-treated L. major infected Balb/c mice), 2: positive control group (GUL200 mg/kg), 3: vehicle control (modified solvent of 10% acetic acid with appropriate pH), 4: B20 mg/kg, 5: BK20 mg/kg and 6: K12.5 mg/kg. Next, they were immersed into 70% ethanol, immediately taken out and the heart blood samples were collected. Also, popliteal lymph node of infected footpad was removed and immersed into sterile cold PBS under a class II laminar flow. Lymph nodes were gently crushed and suspended using Pasteur pipette. Then, the parasite burden was measured using LDA assay on 12 serial dilutions of cells which cultured in 96-well plate (8 well for each dilution) containing Schneider medium supplemented with 12% FBS and 1% Streptomycin/Penicillin antibiotics. In addition, controlled plates had distinct promastigote concentrations. After 3 to 7 days incubation at 26 °C, the number of positive wells (existence of motile parasite) and negative wells (absence of motile parasite) were determined by using invert microscope and the results were analyzed by ELIDA software. The parasite burden was measured after logarithmic calculation of the results^17,26^.

#### Histopathological results of Parasite number in different groups received various formulations by using Giemsa staining in infected footpad, liver, spleen and kidney

The organs of footpad, kidney, spleen and liver of infected animals were harvested, immersed into 10% formalin and paraffinized. Then, the paraffinized tissues were cut at 5 µm thick sections and evaluated histopathologically using Giemsa staining methods to determine the parasite number. The groups of animals were negative control group (non-treated L. major infected Balb/c mice), 2: positive control group (GUL200 mg/kg), 3: vehicle control (modified solvent of 10% acetic acid with appropriate pH), 4: B20 mg/kg, 5: BK20 mg/kg and 6: K12.5 mg/kg. For this purpose, Panoramic Viewer (Software version:1.15) digital microscope (3DHISTECH Ltd., Budapest, Hungary) was used with 40X objective magnification.

#### Statistical analysis

The results of the study were analyzed by one and two-way ANOVA test as well as Prism software and the significance level was set at P < 0.05 considering appropriate post hoc tests.

## Electronic supplementary material

Supplementary figures

## References

[1] Scott, P. & Novais, F. O. Cutaneous leishmaniasis: immune responses in protection and pathogenesis. *Nature Reviews Immunology* (2016). 10.1038/nri.2016.72 27424773

[2] Ramírez, J. D. *et al*. Taxonomy, diversity, temporal and geographical distribution of cutaneous leishmaniasis in Colombia: a retrospective study. *Scientificreports6* (2016). 10.1038/srep28266 PMC4916406 27328969

[3] Van Bocxlaer K, Yardley V, Murdan S, Croft SL (2016). Drug permeation and barrier damage in Leishmania-infected mouse skin. Journal of Antimicrobial Chemotherapy.

[4] Galvão EL, Rabello A, Cota GF (2017). Efficacy of azole therapy for tegumentary leishmaniasis: A systematic review and meta-analysis. PloS one.

[5] Sousa MC (2014). Antileishmanial activity of semisynthetic lupane triterpenoids betulin and betulinic acid derivatives: synergistic effects with miltefosine. PloS one.

[6] Yogeeswari P, Sriram D (2015). Betulinic acid and its derivatives: a review on their biological properties. Current medicinal chemistry.

[7] Meira CS (2016). Antiparasitic evaluation of betulinic acid derivatives reveals effective and selective anti-Trypanosoma cruzi inhibitors. Experimentalparasitology.

[8] Haavikko, R. Synthesis of betulin derivatives with new bioactivities. **5** (2015).

[9] Alakurtti S (2010). Synthesis and anti-leishmanial activity of heterocyclic betulin derivatives. Bioorganic & medicinal chemistry.

[10] Domínguez-Carmona D (2010). Antiprotozoal activity of betulinic acid derivatives. Phytomedicine1.

[11] Chowdhury S (2015). Novel betulin derivatives as antileishmanial agents with mode of action targeting type IB DNA topoisomerase. Molecular pharmacology.

[12] Chowdhury AR (2003). Dihydrobetulinic acid induces apoptosis in Leishmania donovani by targeting DNA topoisomerase I and II: implications in antileishmanial therapy. Molecular Medicine.

[13] Saneja A (2017). Development and evaluation of long-circulating nanoparticles loaded with betulinic acid for improved anti-tumor efficacy. International Journal of Pharmaceutics.

[14] Ribeiro TG (2014). An optimized nanoparticle delivery system based on chitosan and chondroitin sulfate molecules reduces the toxicity of amphotericin B and is effective in treating tegumentary leishmaniasis. International journal of nanomedicine.

[15] Liu Y (2016). Antitumor drug effect of betulinic acid mediated by polyethylene glycol modified liposomes. Materials Science and Engineering: C.

[16] Salah, R. Antileishmanial activities of chitin and chitosan prepared from shrimp shell waste (2015).

[17] Tripathi P, Jaiswal AK, Dube A, Mishra PR (2017). Hexadecylphosphocholine (Miltefosine) stabilized chitosan modified Ampholipospheres as prototype co-delivery vehicle for enhanced killing of L. donovani. International journal of biological macromolecules.

[18] Jain V (2014). Chitosan-assisted immunotherapy for intervention of experimental leishmaniasis via amphotericin B-loaded solid lipid nanoparticles. Applied biochemistry and biotechnology.

[19] Wang JJ (2011). Recent advances of chitosan nanoparticles as drug carriers. International journal of nanomedicine.

[20] Tenzer S (2013). Rapid formation of plasma protein corona critically affects nanoparticle pathophysiology. Nature nanotechnology.

[21] Joseph Y (2003). Self-assembled gold nanoparticle/alkanedithiol films: preparation, electron microscopy, XPS-analysis, charge transport, and vapor-sensing properties. The Journal of Physical Chemistry.

[22] Sreeprasad T, Maliyekkal SM, Lisha K, Pradeep T (2011). Reduced graphene oxide–metal/metal oxide composites: facile synthesis and application in water purification. Journal of hazardous materials.

[23] Belosevic M, Finbloom D, Van Der Meide PH, Slayter M, Nacy C (1989). Administration of monoclonal anti-IFN-gamma antibodies *in vivo* abrogates natural resistance of C3H/HeN mice to infection with Leishmania major. The Journal of Immunology.

[24] Ribeiro, T. G. *et al*. Novel targeting using nanoparticles: an approach to the development of an effective anti-leishmanial drug-delivery system (2014). 10.2147/IJN.S55678 PMC3931713 24627630

[25] Tripathi P (2015). Development of 4-sulfated N-acetyl galactosamine anchored chitosan nanoparticles: A dual strategy for effective management of Leishmaniasis. Colloids and Surfaces B: Biointerfaces.

[26] Singh PK (2017). Chitosan coated PluronicF127 micelles for effective delivery of Amphotericin B in experimental visceral leishmaniasis. International journal of biological macromolecules.

[27] Ahmed TA, Aljaeid BM (2016). Preparation, characterization, and potential application of chitosan, chitosan derivatives, and chitosan metal nanoparticles in pharmaceutical drug delivery. Drug design, development and therapy.

[28] Asthana S (2013). Immunoadjuvant chemotherapy of visceral leishmaniasis in hamsters using amphotericin B-encapsulated nanoemulsion template-based chitosan nanocapsules. Antimicrobial agents and chemotherapy.

[29] Gupta PK (2015). Self assembled ionically sodium alginate cross-linked amphotericin B encapsulated glycol chitosan stearate nanoparticles: applicability in better chemotherapy and non-toxic delivery in visceral leishmaniasis. Pharmaceuticalresearch.

[30] Limpeanchob N, Tiyaboonchai W, Lamlertthon S, Viyoch J, Jaipan S (2013). Efficacy and toxicity of amphotericin B-chitosan nanoparticles in mice with induced systemic candidiasis. Naresuan University Journal: Science and Technology.

[31] Hejazi R, Amiji M (2002). Stomach-specific anti-H. pylori therapy. I: preparation and characterization of tetracyline-loaded chitosan microspheres. International journal of pharmaceutics.

[32] Mohammadzadeh P, Cohan RA, Ghoreishi SM, Bitarafan-Rajabi A, Ardestani MS (2017). AS1411 Aptamer-Anionic Linear Globular Dendrimer G2-Iohexol Selective Nano-Theranostics. Scientific reports.

[33] Ngadiwiyana N (2018). Synthesis of Nano Chitosan as Carrier Material of Cinnamon’s Active Component. Jurnal Kimia Sains dan Aplikasi.

[34] Huynh, C. T. & Lee, D. S. In *Encyclopedia of Polymeric Nanomaterials* 439–449 (Springer, 2015).

[35] Tamizharasi S, Dubey A, Rathi V, Rathi J (2009). Development and characterization of niosomal drug delivery of gliclazide. Journal of Young Pharmacists.

[36] Irby D, Du C, Li F (2017). Lipid–Drug Conjugate for EnhancingDrug Delivery. Molecular pharmaceutics.

[37] Barzegar-Jalali M (2008). Kinetic analysis of drug release from nanoparticles. Journal of Pharmacy and Pharmaceutical Sciences.

[38] Dash M, Chiellini F, Ottenbrite R, Chiellini E (2011). Chitosan—A versatile semi-synthetic polymer in biomedical applications. Progress in polymerscience.

[39] Cole, P., Bishop, J., Beckstead, J., Titus, R. & Ryan, R. Effect of Amphotericin B Nanodisks on Leishmania major Infected Mice. *Pharmaceutica analyticaacta* **5** (2014). 10.4172/2153-2435.1000311 PMC4288788 25584195

[40] Campos-Salinas J (2013). Protective role of the neuropeptide urocortin II against experimental sepsis and leishmaniasis by direct killing of pathogens. The Journal of Immunology.

[41] Corware K (2015). Accelerated healing of cutaneous leishmaniasis in non-healing BALB/c mice using water soluble amphotericin B-polymethacrylic acid. Biomaterials.

[42] Rocha‐Vieira E (2003). Histopathological outcome of Leishmania major‐infected BALB/c mice is improved by oral treatment with N‐acetyl‐l‐cysteine. Immunology.

[43] Crosby EJ, Goldschmidt MH, Wherry EJ, Scott P (2014). Engagement of NKG2D on bystander memory CD8 T cells promotes increased immunopathology following Leishmania major infection. PLoSpathogens.

[44] Bruni N (2017). Nanostructured delivery systems with improved leishmanicidal activity: a critical review. International journal of nanomedicine.

[45] Jain A, Thakur K, Sharma G, Kush P, Jain UK (2016). Fabrication, characterization and cytotoxicity studies of ionically cross-linked docetaxel loaded chitosan nanoparticles. Carbohydrate polymers.

[46] Morakul B, Suksiriworapong J, Chomnawang MT, Langguth P, Junyaprasert VB (2014). Dissolution enhancement and *in vitro* performance of clarithromycin nanocrystals produced by precipitation–lyophilization–homogenization method. European Journal of Pharmaceutics and Biopharmaceutics.

[47] Koch S (2016). Polycarboxylate ethers: The key towards non-toxic TiO 2 nanoparticle stabilisation in physiological solutions. Colloids and Surfaces B: Biointerfaces1.

[48] Abamor ES (2017). Antileishmanial activities of caffeic acid phenethyl ester loaded PLGA nanoparticles against Leishmania infantum promastigotes and amastigotes *in vitro*. Asian Pacific journal of tropicalmedicine.

[49] Vellozo NS (2017). all-Trans retinoic acid Promotes an M1-to M2-Phenotype shift and inhibits Macrophage-Mediated immunity to Leishmania major. Frontiers in immunology.

[50] Marinho FA (2017). The potent cell permeable calpain inhibitor MDL28170 affects the interaction of Leishmania amazonensis with macrophages and shows anti-amastigote activity. Parasitologyinternational.

[51] Jain, K., Verma, A. K., Mishra, P. R. & Jain, N. K. Surface-engineered dendrimeric nanoconjugates for macrophage-targeted delivery of amphotericin B: formulation development and *in vitro* and *in vivo* evaluation. *Antimicrobial agents and chemotherapy* **59**, 2479–2487 (2015). 10.1128/AAC.04213-14 PMC4394771 25645852

[52] Mishra J, Dey A, Singh N, Somvanshi R, Singh S (2013). Evaluation of toxicity & therapeutic efficacy of a new liposomal formulation of amphotericin B in a mouse model. The Indian journal of medical research.

[53] Araujo AP, Giorgio S (2015). Immunohistochemical evidence of stress and inflammatory markers in mouse models of cutaneous leishmaniosis. Archives of dermatological research.

