# Supplementary material for: Novel Nanosized Chitosan-Betulinic Acid Against Resistant Leishmania Major and First Clinical Observation of such parasite in Kidney
Source: Sci Rep. 2018 Aug 6;8:11759. doi: 10.1038/s41598-018-30103-7 (PMC6078985; doi:10.1038/s41598-018-30103-7)
Supplement: Supplementary file 1 — Supplementary figures [file 41598_2018_30103_MOESM1_ESM.docx]

**Novel Nanosized Chitosan-Betulinic Acid Against Resistant Leishmania Major and First Clinical Observation of such parasite in Kidney**

Tahereh Zadeh Mehrizi^1^, Mehdi Shafiee Ardestani^2,^*, Mostafa Haji Molla Hoseini^3^, Ali Khamesipour^4^, Nariman Mosaffa^3^, Amitis Ramezani^1,^*

^1^Department of Clinical Research, Pasteur Institute of Iran, Tehran, Iran

^2^Department of Radiopharmacy, Faculty of Pharmacy, Tehran University of Medical Sciences, Tehran, Iran

^3^Department of Medical Immunology, School of Medicine, Shahid Beheshti University of Medical Sciences, Tehran, Iran

^4^Center for Research and Training in Skin Diseases and Leprosy, Tehran University of Medical Sciences, Tehran, Iran

**First corresponding author:** Amitis Ramezani, Postal Address: Department of Clinical Research, Pasteur Institute of Iran, Tehran, Iran. Phone number: +982164112812, Mobile: +989122101162, Email: [Amitisramezani@hotmail.com](mailto:Amitisramezani@hotmail.com), ametesramezani@gmail.com

**Second corresponding author:** Mehdi Shafiee Ardestani, Postal Address: Department of Radiopharmacy, Faculty of Pharmacy, Tehran University of Medical Sciences, Tehran, Iran. Mobile: +989128267629, Email: [shafeeardestani@tums.ac.ir](mailto:shafeeardestani@tums.ac.ir), [shafieeardestani@gmail.com](mailto:shafieeardestani@gmail.com)


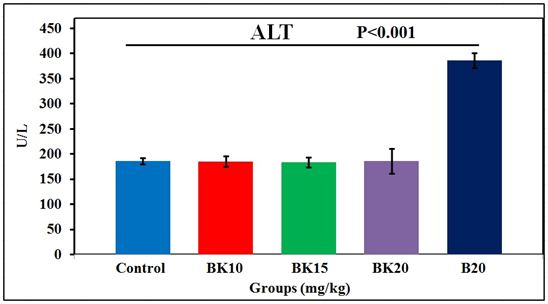

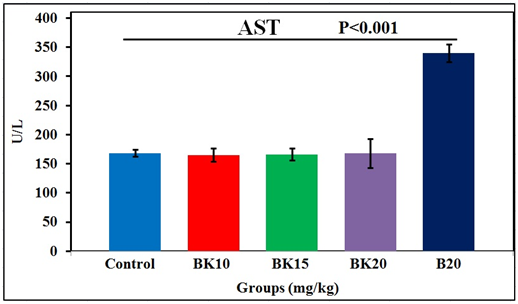

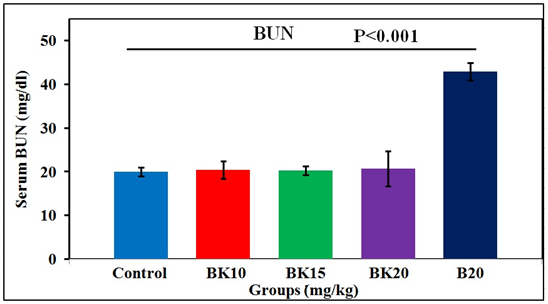

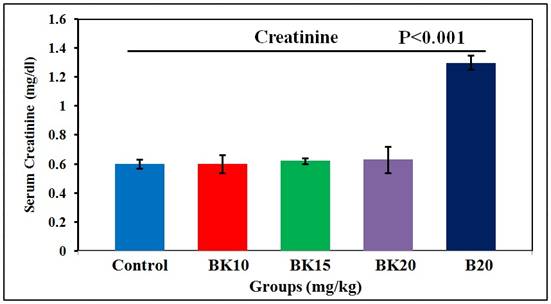

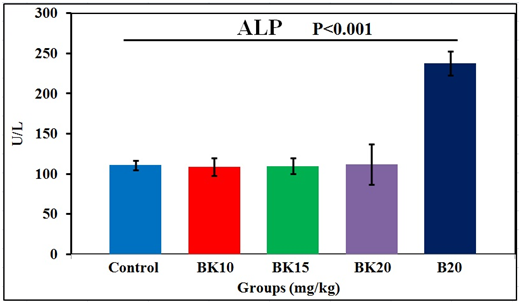


**Supplementary Figure 1. In vivo toxicity evaluation.** The serum concentrations of creatinine, BUN, ALP, AST and ALT in mice receiving BK10, 15, 20 and B20 mg/kg formulations compared to the negative control group. As the figure shows, B has no toxicity when formulated with nanochitosan, while in the free form it is toxic.

**
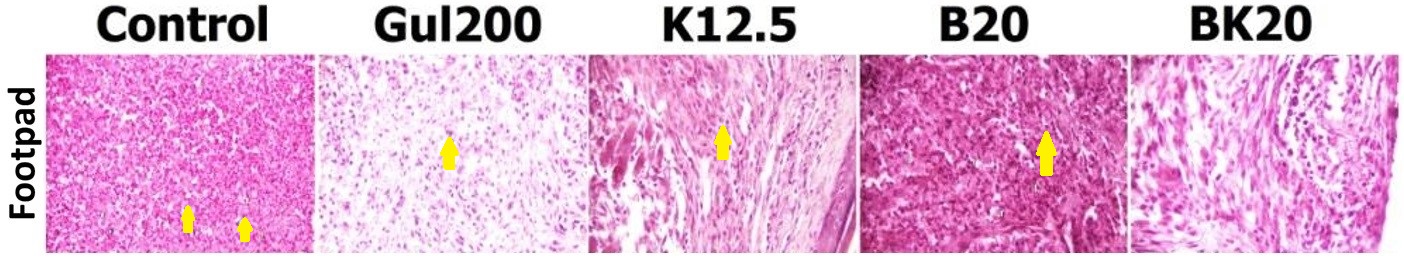
**

**Supplementary Figure 2. Parasite number measurement through histopathological evaluation.** Infected footpad was evaluated through H&E staining for measurement of parasite number in different groups of mice receiving Gul200 mg/kg (positive control group), nanoK12.5 mg/kg, B20 mg/kg and BK20 mg/kg compared to the negative control group (non-treated infected mice). The results showed that there was no parasite in BKB0 mg/kg receiver mice compared to positive and negative control groups.
